# Health facility assessment of small and sick newborn care in low- and middle-income countries: systematic tool development and operationalisation with NEST360 and UNICEF

**DOI:** 10.1186/s12887-023-04495-z

**Published:** 2024-03-07

**Authors:** Rebecca E. Penzias, Christine Bohne, Samuel K. Ngwala, Evelyn Zimba, Norman Lufesi, Ekran Rashid, Edith Gicheha, Opeyemi Odedere, Olabisi Dosunmu, Robert Tillya, Josephine Shabani, James H. Cross, Sara Liaghati-Mobarhan, Msandeni Chiume, George Banda, Alfred Chalira, John Wainaina, David Gathara, Grace Irimu, Steve Adudans, Femi James, Olukemi Tongo, Veronica Chinyere Ezeaka, Georgina Msemo, Nahya Salim, Louise T. Day, Timothy Powell-Jackson, Jaya Chandna, Maureen Majamanda, Elizabeth M. Molyneux, Maria Oden, Rebecca Richards-Kortum, Eric O. Ohuma, Chris Paton, Tedbabe Hailegabriel, Gagan Gupta, Joy E. Lawn, with the Health Facility Assessment Technical Content Reviewers, Co-design Group, Health Facility Assessment Data Collection Learning Group, Aba Asibon, Megan Heenan, Ivan Mambule, Kara Palamountain, Martha Mkony, Kondwani Kawaza, Jenny Werdenberg, Victor Tumukunde, Sue Prullage, Dickson Otiangala, Betsy Asma, Cally Tann, Danica Kumara, Melissa M. Medvedev, Simeon Yosefe, Mike English, Honorati Masanja, Bertha Kaudzu, Angeline Chiotcha, Harriet Ruysen, Oona Campbell, Gina Murphy, Samantha Herrera, Natasha Rhoda, Lily Kak, Vincent Ochieng, Sam Wachira, Catherine Okunola, Olabanjo Okunlola Ogunsola, Adewole, Donat Shamba, Ahazi Manjonda, Irabi Kassim, Giorgia Gon, Grace Soko, Emmie Mbale, Mwanamvua Boga, Charles Osuagwu, Mary Ngugi, Harold Chimphepo, Esan Bukola, Valentino Mvanga, Linda Kagasi, Josephat Mutakyamilwa, Maureen Valle, Carolyne Mwangi, Bridget Wesonga, Audrey Chepkemoi, Joseph Chabi, Mohammed Sheikh, Robert Ngunjiri, Beth Maina, Mary Waiyego, Enock Sigilai, Grace Wasike, Isaac Cheptiany, Josephine Aritho, Josephine Bariu, Lucy Kinyua, Lydia Karimurio, Martin Matingi, Fred Were, Wanjiku Manguyu, Jenny Carns, Caroline Noxon, Esalee Andrade, Taylor Boles, Brady Hunt, Akshaya Santhanaraj, Madeleine Tadros, Meghan B. Kumar, Christina Mchoma, Joseph Bilitinyu, Pius Chalamanda, Mirriam Dzinkambani, Ruth Mhango, Fanny Stevens, Joseph Mulungu, Blessings Makhumula, Loveness Banda, Charles Banda, Brian Chumbi, Chifundo Banda, Evelyn Chimombo, Nicodemus Nyasulu, Innocent Ndau, Pilirani Kumwembe, Edna Kerubo, Nyphry Ambuso, Kevin Koech, Noel Waithaka, Calet Wakhungu, Steven Otieno, Felix Bahati, Josphine Ayaga, Jedida Obure, Nellius Nderitu, Violet Mtambo, George Mkude, Mustapha Miraji, Caroline Shayo, Camilius Nambombi, Christopher Cyrilo, Temilade Aderounmu, Akingbehin Wakeel Wale, Odeleye Victoria Yemisi, Akinola Amudalat Dupe, Samuel Awolowo, Ojelabi Oluwaseun, John Ajiwohwodoma Ovuoraye, Balogun Adeleke Mujaid, Adedoyin Fetuga, Juilana Okanlawon, Flora Awosika, Awotayo Olasupo Michael, Omotayo Adegboyega Abiodun

**Affiliations:** 1 Maternal, Adolescent, Reproductive, & Child Health Centre, London School of Hygiene & Tropical Medicine, London, UK; 2 Rice360 Institute for Global Health Technologies, Rice University, Texas, USA; 3 Ifakara Health Institute, Ifakara, Tanzania; 4 Research Support Center, School of Public Health and Family Medicine, Kamuzu University of Health Sciences, Blantyre, Malawi; 5 Ministry of Health, Lilongwe, Malawi; 6 Aga Khan University Hospital, Nairobi, Kenya; 7 APIN Public Health Initiatives, Abuja, Nigeria; 8 Product Innovation Centre, United Nations Children’s Fund, Blantyre, Malawi; 9 Department of Paediatrics, Kamuzu University of Health Sciences (Formerly College of Medicine, University of Malawi), Blantyre, Malawi; 10 Department of Paediatrics, Kamuzu Central Hospital, Lilongwe, Malawi; 11 Kamuzu University of Health Sciences, Blantyre, Malawi; 12 Kenya Medical Research Institute (KEMRI)-Wellcome Trust, Nairobi, Kenya; 13 Department of Paediatrics and Child Health, University of Nairobi, Nairobi, Kenya; 14 Academy for Novel Channels in Health and Operations Research (ACANOVA) Africa, Nairobi, Kenya; 15 Newborn Branch, Federal Ministry of Health, Abuja, Nigeria; 16 FWACP Department of Paediatrics, College of Medicine, University of Ibadan, Ibadan, Nigeria; 17 Department of Paediatrics, College of Medicine, University of Lagos, Lagos, Nigeria; 18 Department of Health Systems, Impact Evaluation and Policy, Ifakara Health Institute, Dar Es Salaam, Tanzania; 19 Ministry of Health and Social Welfare, Dar Es Salaam, Tanzania; 20 Department of Paediatrics and Child Health, Muhimbili University of Health and Allied Sciences, Dar Es Salaam, Tanzania; 21 Maternal and Newborn Health Group, Department of Infectious Disease Epidemiology, London School of Hygiene & Tropical Medicine, London, UK; 22 Department of Global Health and Development, London School of Hygiene & Tropical Medicine, London, UK; 23 Kamuzu University of Health Sciences (Formerly Kamuzu College of Nursing, University of Malawi), Blantyre, Malawi; 24 Centre for Tropical Medicine and Global Health, Nuffield Department of Medicine, University of Oxford, Oxford, UK; 25 Department of Information Science, University of Otago, Dunedin, New Zealand; 26 Program Group, Health Programme UNICEF Headquarters, New York, USA

**Keywords:** Newborn, Low- and Middle-Income Countries, Inpatient Care, Service readiness, Health facility assessment, ENAP coverage targets, Level-2 small and sick newborn care

## Abstract

**Background:**

Each year an estimated 2.3 million newborns die in the first 28 days of life. Most of these deaths are preventable, and high-quality neonatal care is fundamental for surviving and thriving. Service readiness is used to assess the capacity of hospitals to provide care, but current health facility assessment (HFA) tools do not fully evaluate inpatient small and sick newborn care (SSNC).

**Methods:**

Health systems ingredients for SSNC were identified from international guidelines, notably World Health Organization (WHO), and other standards for SSNC. Existing global and national service readiness tools were identified and mapped against this ingredients list. A novel HFA tool was co-designed according to a priori considerations determined by policymakers from four African governments, including that the HFA be completed in one day and assess readiness across the health system. The tool was reviewed by > 150 global experts, and refined and operationalised in 64 hospitals in Kenya, Malawi, Nigeria, and Tanzania between September 2019 and March 2021.

**Results:**

Eight hundred and sixty-six key health systems ingredients for service readiness for inpatient SSNC were identified and mapped against four global and eight national tools measuring SSNC service readiness. Tools revealed major content gaps particularly for devices and consumables, care guidelines, and facility infrastructure, with a mean of 13.2% (*n* = 866, range 2.2–34.4%) of ingredients included. Two tools covered 32.7% and 34.4% (*n* = 866) of ingredients and were used as inputs for the new HFA tool, which included ten modules organised by adapted WHO health system building blocks, including: infrastructure, pharmacy and laboratory, medical devices and supplies, biomedical technician workshop, human resources, information systems, leadership and governance, family-centred care, and infection prevention and control. This HFA tool can be conducted at a hospital by seven assessors in one day and has been used in 64 hospitals in Kenya, Malawi, Nigeria, and Tanzania.

**Conclusion:**

This HFA tool is available open-access to adapt for use to comprehensively measure service readiness for level-2 SSNC, including respiratory support. The resulting facility-level data enable comparable tracking for *Every Newborn* Action Plan coverage target four within and between countries, identifying facility and national-level health systems gaps for action.

**Supplementary Information:**

The online version contains supplementary material available at 10.1186/s12887-023-04495-z.

## Key findings

**Table:** 

**1. WHAT WAS KNOWN?**
• Over 100 countries are implementing the *Every Newborn* Action Plan (ENAP), but there are no standardised health facility assessment (HFA) tools to assess progress towards ENAP coverage target four for 80% of districts in every country to have at least one level-2 small and sick newborn care (SSNC) unit by 2025 • Existing tools measuring health facility service readiness do not evaluate level-2 SSNC meeting World Health Organization (WHO) standards, and there are notable gaps for Continuous Positive Airway Pressure (CPAP) service readiness, which is more complex but important to meeting the Sustainable Development Goal for neonatal mortality reduction
**2. WHAT WAS DONE THAT IS NEW?**
• We applied a systematic, evidence-based approach in three steps to review, co-design, and operationalise a HFA for SSNC • Step 1. Review: A list of 866 key health systems ingredients for SSNC was established from existing SSNC guidelines from WHO and expert review, building off Moxon et al*.'s* list of 654 key ingredients for SSNC. Twelve existing service readiness tools, including for maternal and newborn health and general service provision, were identified and mapped against the list of 866 key ingredients for SSNC • Step 2. Co-Design: A new health facility assessment tool measuring service readiness for SSNC was developed to meet a priori criteria set by governments and partners in the Newborn Essential Solutions and Technologies (NEST360) alliance using the list of key ingredients for SSNC and adapting from previous tools • Step 3. Operationalise: The tool was refined through data collection and learnings at 64 hospitals in Kenya, Malawi, Nigeria, and Tanzania
**3. WHAT WAS FOUND?**
• Step 1. Review: Identified health facility assessment tools included a mean of 13.2% of key ingredients for SSNC (*n* = 866, range 2.2—34.4%), with the Emergency Obstetric and Newborn Care (EmONC) and Every Preemie-SCALE facility assessment for inpatient care of the newborn and young infant covering 32.7% and 34.4% (*n* = 866), respectively. Hence, a new tool was required • Step 2. Co-design: A new HFA tool was co-designed with ten discrete modules organised by adapted WHO Health System Building Blocks (HSBBs), including components on infrastructure, pharmacy and laboratory, medical devices and supplies, biomedical technician workshop, human resources, information systems, leadership and governance, family-centred care, and infection prevention and control • Step 3. Operationalise: the HFA was possible to complete in one day per hospital by a team of trained Ministry of Health assessors. Learnings around HFA team structure, communication, and logistics were incorporated into later rounds
**4. WHAT NEXT?**
• This novel HFA tool for SSNC is open access and available for adaptation and use in other countries for tracking progress towards the ENAP coverage target for level-2 SSNC. Moving the tool from REDCap to an open access platform is planned to enable wider use. The resultant data can help identify facility and national-level health systems gaps, which, if addressed, can improve the quality of newborn care • Multi-country HFA analyses have the potential for tracking health facility service readiness over time and examining associations of health systems summary scores (e.g., by HSBB) with newborn outcomes • There is interest in developing additional modules, such as to assess level-3 SSNC

## Background

To assess progress towards the *Every Newborn* Action Plan (ENAP) coverage target for level-2 newborn care units, we need a health facility assessment tool to measure facility readiness to provide level-2 small and sick newborn care (SSNC), including Continuous Positive Airway Pressure (CPAP) [1]. The ENAP countries and partners with the Ending Preventable Maternal Mortality (EPMM) community set joint coverage targets and milestones for 2020–2025, including coverage of antenatal, birth, postnatal and newborn care at national and sub-national levels, in addition to the fourth ENAP coverage target for SSNC for 80% of districts in every country to have at least one level-2 inpatient newborn care unit including CPAP by 2025 [1]. Until now there has been no standardised assessment for countries to measure their progress towards this coverage target.

Health facility service readiness refers to the capacity of health facilities to provide high-quality care. Service readiness tools often focus on assessing the availability of items and components needed to provide clinical interventions, or signal functions, to improve survival [2–4]. They are widely used for measuring basic and comprehensive emergency obstetric care and wider service provision [2, 5–10]. Some of these tools include assessment of readiness to provide immediate and essential newborn care, including cord care and warming, especially in maternity services [2, 5]. Some countries, including India, Bangladesh, Sierra Leone, and Ghana, use these global service readiness tools to assist in planning health services and resource allocation for newborn care [11–14]. There is currently no consensus around signal functions for level-2 SSNC, though ongoing revisions to the emergency obstetric care (EmOC) monitoring framework aim to include these newborn signal functions [11, 15]. However, existing tools do not comprehensively assess service readiness for SSNC and have gaps in assessing level-2 care, including for respiratory support, notably CPAP and other medical devices, and for other interventions requiring medically complex care with highly skilled nursing support [1, 16]. To fill this gap, a health facility assessment (HFA) tool was systematically developed and co-designed with four African governments to assess service readiness for level-2 SSNC. This tool builds on existing tools and was designed to complement, rather than replace, these existing service readiness tools. The tool design was facilitated by Newborn Essential Solutions and Technologies (NEST360), a multi-country alliance, including four African governments, aiming to reduce deaths amongst inpatient newborns from 2019 to 2023, in partnership with United Nations Children's Fund (UNICEF) and other key stakeholders. The HFA tool was developed alongside other global public goods, including NEST360 clinical education modules, a standardised neonatal inpatient dataset, and a publicly available toolkit for SSNC [17–19].

Feasibility of implementation of this service readiness tool was a key factor in tool design. A priori considerations for feasibility were jointly identified by country governments and implementers, including designing a tool that can be completed in just one day at a hospital and including assessment of ingredients across the health system. These a priori considerations were addressed and incorporated throughout the design stages.

### Aim

This paper is part of a supplement reporting findings and learnings from NEST360, an alliance of partners, including four African governments (Kenya, Malawi, Nigeria, and Tanzania), working to reduce neonatal inpatient deaths by improving level-2 newborn care in hospitals. In this paper, we aim to describe the systematic, evidence-based development and operationalisation of a health facility assessment tool for service readiness for level-2 SSNC in low- and middle-income countries. Specifically, we cover the following three objectives:

- **Objective 1:** Review existing standards and establish a list of ingredients for level-2 SSNC; scope and map tools measuring service readiness against ingredients for SSNC
- **Objective 2:** Co-design content of a novel HFA tool according to a priori considerations and undertake review by global experts
- **Objective 3:** Refine and operationalise the HFA tool in 64 hospitals in Kenya, Malawi, Nigeria, and Tanzania

## Methods

**Fig. 1:**
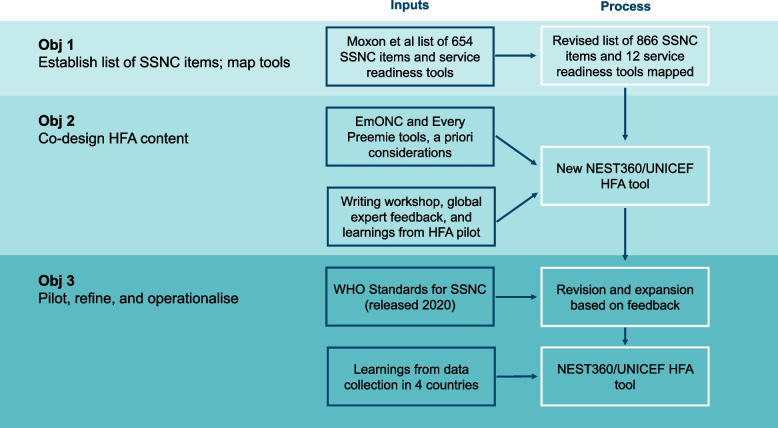
Flow diagram of NEST360/UNICEF Health Facility Assessment tool systematic development stages and process. Abbreviations: NEST360, Newborn Essential Solutions and Technologies; UNICEF, United Nations Children's Fund; HFA, Health Facility Assessment; SSNC Small and Sick Newborn Care; EmONC, Emergency Obstetric and Newborn Care; WHO, World Health Organization

The HFA tool, facilitated by NEST360, was systematically developed using a three-step evidence-based process summarised in Fig. 1 and adapted from a recognised user-design process [20] (Additional file 1). The tool was developed through an iterative process from June 2019 through March 2021, including refinement and operationalisation at 64 hospitals implementing with NEST360.

### Methods by objectives

#### Objective 1: Review existing standards and establish a list of ingredients for level-2 SSNC; scope and map tools measuring service readiness against ingredients for SSNC

Moxon et al. developed a list of 654 key health systems ingredients for service readiness for inpatient SSNC [16]. The ingredients were identified from existing international guidelines and standards for SSNC focusing on drugs, devices and consumables, and clinical management and treatment protocols. We expanded this list to include additional health systems ingredients and components necessary for providing care for each of the World Health Organization (WHO) signal functions for newborn care [21]. Additional ingredients primarily included consumables and spare parts for devices that are necessary for providing level-2 SSNC.

**Fig. 2:**
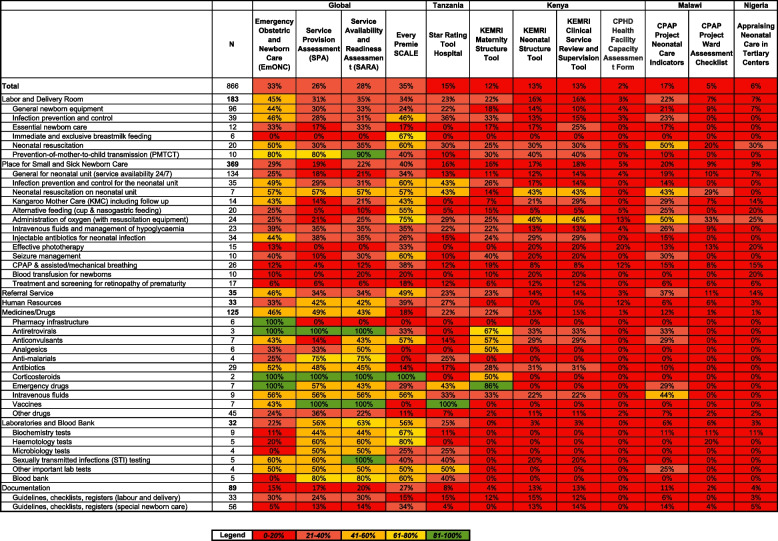
Heatmap of the percentage of key ingredients for small and sick newborn care (*n* = 866) collected in select service readiness tools. Abbreviations: SCALE, Scaling, Catalyzing, Advocating, Learning, and Evidence-driven; KEMRI, Kenya Medical Research Institute; CPHD, Center for Public Health and Development; CPAP, Continuous Positive Airway Pressure

Existing national tools for the four countries implementing with NEST360 and more widely used global tools that measure hospital service readiness for SSNC used in multiple countries and available online were identified by a scoping review and through global experts and mapped against the expanded list of key ingredients for SSNC. Individual questions from each tool were considered to cover the relevant ingredient if the question was an exact or partial match for the ingredient. Heatmaps were developed to demonstrate the proportion of key ingredients for SSNC included in existing service readiness tools (Fig. 2). The WHO standards for SSNC were not yet available at this stage [22].

#### Objective 2: Co-design content of new HFA tool according to a priori considerations and undertake review by global experts

A priori design considerations were based on the four country governments and members of the NEST360 alliance involved during this stage of development (Table 1). These stakeholders noted that it was vital for a new HFA tool to link to existing service readiness tools for maternal and obstetric care, and to be feasible, notably done in just one day to plan for sustainability of the tool and for hospitals to receive timely reports of results.

**Table 1:** A priori considerations for development and use of a health facility assessment tool measuring service readiness for small and sick newborn care

• *Remit*: Focused on level-2 small and sick newborn care and in support of World Health Organization (WHO) standards for improving the quality of care for small and sick newborns
• *Ability to integrate*: Complementary to existing service readiness tools, so can be used as a module with other tools, e.g. maternal and obstetric care (e.g. EmONC) and child health care Health Facility Assessments
• *Structure*: Organised by WHO health system building block linking to the NEST360 Theory of Change
• *Feasibility*: Possible to conduct at a hospital in one day by a multi-disciplinary team
• *Data use*: Sharing structured reports with ward and hospital management to act on identified gaps and areas of good performance

*Abbreviations:* *NEST360* Newborn Essential Solutions and Technologies, *EmONC* Emergency Obstetric and Newborn Care

Mapping existing tools against the expanded list of key ingredients for SSNC identified important gaps in assessment of service readiness for inpatient SSNC. Tools with the highest proportion of ingredients were used as a starting point to develop the new HFA tool [2]. The new tool was structured to align with the HSBB framework adapted from WHO [23]. Health financing, an important component of health systems, was not included as it was believed that health financing would be better addressed at the subnational rather than facility level. Observation and interviewer-led assessment were selected for their appropriateness in measuring relevant ingredients and for ease of data collection given a priori criteria limiting HFA data collection to one day. Direct observation was used to observe physical items and staff practices. Interviewer-led assessments with hospital staff were used to assess other constructs with verification of responses through review of available documentation.

Initial tool co-design was guided by a multi-day writing workshop (June 2019) with 18 multi-disciplinary stakeholders and experts, including data managers, study implementers, clinicians, nurses, and Ministry of Health officials (i.e., from information systems departments) from Kenya, Malawi, Nigeria, and Tanzania. During the workshop, new questions were written to cover gaps in existing tools using the expanded list of key ingredients. The draft tool developed during the workshop was subsequently shared with other multi-disciplinary global experts from 16 institutions within the NEST360 alliance, and feedback was systematically included.

The tool was reviewed by multidisciplinary global experts external to the NEST360 alliance, including WHO and UNICEF. Feedback was provided by email and incorporated by revising or adding new questions. Additional line-by-line reviews with experts, including many mid-level and senior Ministry of Health staff, took place in Kenya, Malawi, Nigeria, and Tanzania. The WHO standards for improving the care of small and sick newborns in health facilities were released in September 2020 [22]. A line-by-line comparison of the WHO standards and the new NEST360/UNICEF HFA tool was conducted, and gaps in the new HFA tool were addressed.

#### Objective 3: Refine and operationalise the HFA tool in 64 hospitals in Kenya, Malawi, Nigeria, and Tanzania

The tool was pilot tested using paper data collection at one primary and one secondary hospital in Malawi by senior Ministry of Health staff, including a line-by-line review. After the initial pilot visits and feedback, the paper tool was refined and coded into REDCap to streamline data collection and improve data quality [24]. Subsequently, the electronic REDCap tool was used for data collection at further pilot visits conducted on tablets at 11 primary, secondary, and tertiary hospitals in Kenya and Malawi. Before each pilot visit, trainings were conducted by Ministry of Health staff in Kenya and Malawi and other key stakeholders with expertise in clinical care, laboratory and pharmacy, biomedical device management and maintenance, human resources, and health and data systems. During these trainings, additional line-by-line reviews of the tool were conducted. Practical considerations and learnings from pilot visits and assessor feedback were also incorporated into the tool implementation and visit processes.

Synthesising feedback from pilot learnings and review led to restructuring of the tool to improve visit flow during data collection and better align with the HSBBs, and address additional gaps identified. A section was added to assess the biomedical workshop infrastructure and preventive and corrective maintenance of devices. Laboratory capacity for microbiology assessment was expanded to align with the Microbiology Investigation Criteria for Reporting Objectively (MICRO) framework [25]. A hand hygiene observation component adapted from the WHO tool was incorporated [26]. Medical records storage systems were included to assess how records are stored and used during and after hospitalisation [27]. These additional sections were developed by adapting existing WHO and other global tools (where possible) and through review with experts [28–31].

Each HFA team included Ministry of Health assessors at the national and district levels and a team supervisor, who was responsible for coordination and support (Table 2). Training was designed for four days involving assessors with the following expertise: nursing and other clinical, engineering, laboratory, pharmacy, and health information systems.

**Table 2:** Health facility assessment data collection multi-disciplinary team composition and background

	**Background**	**Assigned HFA Modules**
**Assessors**	Clinician with familiarity with facility and infection prevention infrastructure	• Facility Infrastructure • Leadership and Governance • Hand Hygiene
	Clinician with familiarity with neonatal unit and infection prevention infrastructure	• Neonatal Unit Infrastructure • Hand Hygiene
	Laboratory specialist with familiarity with laboratory equipment	• Laboratory and Pharmacy • Hand Hygiene
	Biomedical technician/engineer with familiarity with general and neonatal-specific medical supplies	• Medical Devices and Supplies • Hand Hygiene
	Biomedical technician/engineer with familiarity with biomedical workshop tools and spare parts	• Biomedical Workshop • Hand Hygiene
	Clinician with familiarity with neonatal unit	• Human Resources • Family-Centred Care • Hand Hygiene
	Information systems specialist with familiarity with hospital forms and registers	• Information Systems • Hand Hygiene
**Supervisor**	Familiarity with newborn care, experience with health facility assessments	• Not applicable

HFA data collection was completed in one day at each hospital, as requested by country governments during the co-design phase, using the mobile REDCap application on Android tablets [24]. There was variability in the numbers of hours for each HFA notably driven by size and layout of hospital, responsiveness and availability of management, and availability of key respondents. Data quality was verified by HFA team supervisors and the NEST360 country database manager. All HFA data were synced to and stored on servers of the designated country partner during and after data collection. Data quality, cleaning and analysis scripts were developed in Stata version 17™ (StataCorp LLC, Texas, USA) and R (R Foundation for Statistical Computing, Vienna, Austria) software.

## Results

### Results by objective

#### Objective 1: Review existing standards and establish a list of ingredients for level-2 SSNC; scope and map tools measuring service readiness against ingredients for SSNC

The initial list of 654 key health systems ingredients for service readiness for inpatient SSNC from Moxon et al was re-organised by HSBB to reduce repeating ingredients per intervention, and then expanded to 866 key ingredients [16]. The 212 additional ingredients particularly focus on device consumables and spare parts. A complete list of ingredients can be found in Additional file 2.

Four global and eight national tools measuring service readiness for SSNC were identified (Table 3). Tools included a mean of 13.2% (*n* = 866, range 2.2–34.4%) of ingredients from the list of key ingredients for SSNC (Fig. 2). Global tools assessed availability and functionality of many key ingredients for medicines, laboratory testing and supplies; however, global tools had gaps in assessment of devices and consumables, care guidelines, and facility infrastructure. The EmONC and Every Preemie-SCALE tools were found to cover 32.7% and 34.4% (*n* = 866) of ingredients for SSNC and were used as a starting point to develop a new HFA tool [2] (Fig. 2). All newborn care components from the EmONC were included, and some maternal components of the EmONC were adapted for newborn care. Questions were also adapted from the Every Preemie-SCALE tool [32]. The mapping is detailed in Additional file 3.

**Table 3:** Existing service readiness tools including small and sick newborn care content scoped

	**Tool name**	**Source**	**Purpose**
**Global**	Service Provision Assessment (SPA)	United States Agency for International Development -Demographic and Health Surveys	To provide a comprehensive overview of a country's health service delivery
	Service Availability and Readiness Assessment (SARA)	World Health Organization (WHO)	To assess on a regular basis service delivery (availability and readiness) for all health programmes
	Emergency Obstetric and Newborn Care (EmONC)	United Nations Population Fund / Averting Maternal Death and Disability (UNFPA / AMDD)	To assess readiness for obstetric signal functions
	Every Preemie-SCALE Facility Assessment for Inpatient Care of Small and Sick Newborn	Every Preemie- SCALE	To provide information on the current situation for newborns and young infants in a country
**Kenya**	Clinical Service Review and Supervision Tool—Quality of Neonatal Hospital Care	Kenya Medical Research Institute (KEMRI)	An audit of neonatal care services provided by clinical training centres was undertaken to identify areas requiring improvement as part of wider efforts to improve newborn survival in Kenya
	Maternity Structure Tool	Kenya Medical Research Institute (KEMRI)	To determine the quality of comprehensive emergency obstetric care, through the lens of clinical documentation of process indicators of selected emergency obstetric conditions that mostly cause maternal mortality on admission to the labour ward
	Neonatal Structure Tool	Kenya Medical Research Institute (KEMRI)	Part of the Clinical Information Network Project to improve small and sick newborn care
	Health Facility Capacity Assessment Form	Center for Public Health and Development (CPHD)	Part of the Center for Public Health and Development Project to assess facility capacity
**Malawi**	Neonatal Care Indicators Checklist	CPAP quality improvement program (QIP)	Part of the Ministry of Health and Rice360 CPAP scale-up project
	Ward Assessment Checklist	CPAP quality improvement program (QIP)	
**Tanzania**	Star Rating Assessment: Hospital, Health Center and Dispensary	Ministry of Health	Ministry of Health, facility rating system
**Nigeria**	Appraising Neonatal Care in Tertiary Centers	Lagos University Teaching Hospital	To be used for a Nigerian Neonatal registry documenting patient and institutional level clinical information essential to understanding both beneficial practices that can be shared, and drivers of poor outcomes that can be improved upon in neonatal intensive care units (NICUs) in Nigeria

*Abbreviations*: *SCALE* Scaling, Catalyzing, Advocating, Learning, and Evidence-driven, *CPAP* Continuous Positive Airway Pressure

#### Objective 2: Co-design content of novel HFA tool according to a priori considerations and undertake review by global experts

Expert review of the tool was incorporated throughout the tool development, both internally and externally, which included restructuring the tool, and revising or adding questions and answer options. Reviewers (*n* = 150) represented many different organisations, including local and national ministries of health, health facilities, non-governmental organisations, United Nations and other international organisations, and academic universities. Reviewers were organised by their institutional affiliation (i.e., government) and discipline (i.e., clinical). Reviewers had expertise in clinical care (*n* = 67, 45%), engineering (*n* = 30, 20%), laboratory and pharmacy (*n* = 7, 5%), health systems and health programmes (*n* = 26, 17%), and data systems (*n* = 20, 13%). Nearly half of reviewers were from governments, and about a third from academia.

#### Objective 3: Refine and operationalise the HFA tool in 64 hospitals in Kenya, Malawi, Nigeria, and Tanzania

Practical considerations and learnings from pilot visits and assessor feedback were grouped into three key themes: team structure, communication, and logistics (Table 4). For team structure, it was important to select data collectors with specific backgrounds and strengths to collect high-quality data. Communication was identified early as a key part of visit success. Informing facility staff, including facility management, department heads, and anyone who may be interviewed during the visit, in advance was essential. Logistics learnings included requesting an off-duty nurse employed at the hospital to be present and provide support in the neonatal unit during the visit, and bringing key items, such as an introduction letter and mobile Wi-Fi. A complete list of recommended equipment can be found in Additional file 4. We also learned that it was important to allow flexibility in selecting who was interviewed during the visit, particularly given different roles and responsibilities across facility levels and countries.

**Table 4:** Learnings from health facility assessment pilot visits and implementation through assessor feedback

Team Structure	Communication	Logistics
• Designate a separate team leader to oversee the visit and provide support • Designate a dedicated person to provide tablet and technical support if collecting data electronically	• Inform facility management and each department covered in assessment in advance of the visit by multiple methods (e.g. formal letter, phone) • Collect staff phone numbers for follow-up questions after the visit • Allow flexibility in respondents during the visit	• Request renumerated off-duty nurse to be present during the visit • Bring key items on the day of the visit, including team leader checklist, mobile Wi-Fi, charging cables, introduction letter, ruler, measuring tape, light meter, oxygen analyser, name tags, and bags • Allow time for travel and delays between visits

The refined version of the new HFA tool includes ten discrete modules which are aligned with the adapted health system building blocks, including the following areas: 1) facility infrastructure; 2) neonatal unit infrastructure; 3) pharmacy and laboratory; 4) medical devices and supplies; 5) biomedical technician workshop; 6) human resources; 7) information systems; 8) leadership and governance; 9) family-centred care; and 10) hand hygiene observation. Topics covered in each module can be found in Table 5, and a more detailed summary of content can be found in Additional file 5. A team of seven external assessors can complete these modules in one day. Two assessors should have familiarity with facility and neonatal ward infrastructure, and infrastructure for infection prevention. Two assessors should have biomedical technology or engineering backgrounds and be familiar with general medical supplies, biomedical workshop tools, and spare parts. The other three assessors should have a laboratory background, a clinical background and ability to conduct a basic clinical knowledge assessment, and a good understanding of hospital forms, registers, and information systems respectively (Table 2).

**Table 5:** NEST360/UNICEF health facility assessment tool categories and sub-categories

HFA modules and components	Description
1A. Facility Infrastructure	Facility Identification Facility Infrastructure Physical areas Autoclave and sterilisation Communication for referral infrastructure Transportation
1B. Neonatal Infrastructure	Neonatal unit infrastructure Neonatal unit infection prevention and control Neonatal unit electricity Neonatal unit layout Admission and referral criteria
2A. Pharmacy and Laboratory	Pharmacy Laboratory equipment, supplies, and testing Laboratory capacity for microbiology Laboratory linkage to neonatal unit Blood bank Neonatal unit medicines
2B. Medical Devices and Supplies	Infection prevention supplies Inventory and forecasting of consumables Neonatal care devices and supplies Maintenance and repair
2C. Biomedical Workshop	Spare parts and tools Storage and layout Other workshop questions Maintenance and repair
3. Human Resources	Facility and neonatal staffing Facility policies and working conditions Clinical care guidelines Newborn care signal functions Supervisory support and motivation
4. Information Systems	Data sources (forms, registers) Filing systems Neonatal data clerks Summary data for reporting Maternal perinatal death surveillance and response Civil registration and vital statistics Electronic infrastructure Indicator variables
5. Leadership and Governance	Target setting Financing reports Staff absenteeism and performance review Clinical audit and management meetings
6. Family-Centred Care	Policies and training Family satisfaction Family involvement Infrastructure
7. Hand Hygiene Observation	Hand hygiene indications and actions

*Abbreviations*: *NEST360* Newborn Essential Solutions and Technologies, *UNICEF* United Nations Children’s Fund

The complete set of tools can be found on the publicly available toolkit for SSNC [17]. These paper tools can be used to conduct the HFA. Additional files 6 and 7 include summary and detailed reporting templates that can be generated and shared with facilities after HFA data collection.

## Discussion

Standardised and comparable HFA data is important for improving service readiness at facility level and tracking and planning provision of care, especially across a whole country. A standard HFA tool for SSNC, focused on the contexts where most neonatal deaths occur, was lacking. Existing tools did not include even half of the necessary health systems ingredients and did not match a priori design criteria from government users to enable sustainable use in routine systems. Therefore, we applied a systematic, evidence-based approach in three steps to design, develop, and operationalise a novel HFA tool, which is now available for adaptation and use to track progress for ENAP targets [17].

We found that existing service readiness tools did not cover most of key ingredients for level-2 SSNC, particularly devices and consumables, clinical management and treatment protocols, and facility infrastructure. The new NEST360/UNICEF HFA tool is distinct from other service readiness tools, focusing on level-2 SSNC including CPAP, which is a major gap in existing service readiness tools [33]. The EmONC and Every Preemie-SCALE tools covered 32.7% and 34.4% (*n* = 866), respectively, of the required ingredients and relevant components from these were incorporated into the new HFA tool [2, 32]. HFA tools for other health services areas or for the wider system remain vital, and this new HFA tool focusing on SSNC can complement these tools. This HFA tool is intended to link as a module with those existing service readiness tools that measure emergency obstetric care on the maternity ward or assess wider health service provision [2, 5, 6].

This NEST360/UNICEF HFA tool is the first to comprehensively measure service readiness for SSNC. One benefit of the tool is that it can be adapted to suit different purposes. For example, individual modules (e.g., laboratory capacity for microbiology, biomedical workshop) can be omitted to save time or used independently, such as the biomedical workshop module, which assesses readiness for repair and maintenance of medical devices that directly impacts care [28]. The tool also provides some questions relevant to level-3 care, and there is scope to expand to an in-depth level-3 SSNC assessment tool.

Strengths of the development process include collaborative co-creation of the tools with four national governments and NEST360 partner organisations, and the inclusive co-design approach with a priori design considerations set by users. In addition, the tool aligns with WHO standards for improving the quality of care for small and sick newborns in health facilities and other WHO guidance where applicable, and was reviewed by more than 150 global and national experts and stakeholders across a range of disciplines [22, 34]. Refining and operationalising at 64 hospitals in Kenya, Malawi, Nigeria, and Tanzania was instrumental, and questions and answer options can now be considered suitable for these contexts. Linked reporting templates can be used by governments to identify health systems gaps and assist in planning health services and resource allocation for newborn care.

There were also limitations. Our review may have missed other tools if not published or open access. The NEST360/UNICEF HFA tool deliberately focuses on comprehensive assessment of service readiness to provide level-2 inpatient SSNC, so an in-depth assessment of readiness to provide level-3 SSNC was considered beyond scope. It was also outside the remit of this tool development to interview parents/guardians or families about their perspectives or to conduct an in-depth assessment of staff clinical skills to assess patient-level clinical care quality, though a high-level assessment of staff clinical skills is included. The co-development and refining occurred in hospitals in Kenya, Malawi, Nigeria, and Tanzania, and may need further adaptations to be suitable in other countries and contexts. As for all assessments of service readiness, it is possible to score highly, but for this not to be associated with improved patient outcomes, such as reduced neonatal mortality.

Neonatal deaths now represent almost half of under-five child deaths; hence, WHO, UNICEF, and other global organisations, including the Network for Improving Quality of Care for Maternal, Newborn, and Child Health (the QOC network), are increasingly focused on improving newborn survival and long-term outcomes [35]. Global targets support this focus. For example, Sustainable Development Goal 3.2 aims that every country reduce newborn deaths to less than 12 deaths per 1000 live births by 2030 [36]. The fourth ENAP coverage target for SSNC aims for 80% of districts in every country to have at least one level-2 inpatient newborn care unit by 2025. To achieve these global targets, hospitals must be ready to provide high-quality care for small and sick newborns, and these services must be geographically distributed and accessible to all mothers and newborns who need them [1, 37, 38]. To save lives and accelerate progress in countries, these service readiness data must be used to identify and close health systems gaps, drive investments, and improve outcomes for every newborn and family in every country.

## Supplementary Information

**Additional file 1. **Evidence-based framework for co-design of mHealth tools and application to the NEST360 design process.

**Additional file 2. **List of 866 key health system ingredients for SSNC.

**Additional file 3. **Health facility assessment mapping dataset.

**Additional file 4. **Recommended equipment for health facility assessments for SSNC.

**Additional file 5. **NEST360/UNICEF health facility assessment content summary.

**Additional file 6. **Health facility assessment summary report template.

**Additional file 7. **Health facility assessment full report template.

**Additional file 8. **Local ethical approval for the complex evaluation of the implementation of a small and sick newborn care package with NEST360.

## Data Availability

Data sharing and transfer agreements were jointly developed and signed by all collaborating partners in the NEST360 alliance. The pooled summary table data generated during the current study are available in Additional file 3. The HFA tool, data dictionary, and associated materials are available from the NEST360/UNICEF Implementation. Toolkit for Small and Sick Newborn Care and NEST360 website [17, 18].

## Abbreviations

CPAP: Continuous Positive Airway Pressure
EmOC: Emergency Obstetric Care
EmONC: Emergency Obstetric and Newborn Care
ENAP: Every Newborn Action Plan
EPMM: Ending Preventable Maternal Mortality
HFA: Health Facility Assessment
HSBB: Health System Building Block
NEST360: Newborn Essential Solutions and Technologies
SCALE: Scaling, Catalyzing, Advocating, Learning, and Evidence-driven
SSNC: Small and Sick Newborn Care
UNICEF: United Nations Children's Fund
WHO: World Health Organization
