# Supplementary material for: Health facility assessment of small and sick newborn care in low- and middle-income countries: systematic tool development and operationalisation with NEST360 and UNICEF
Source: BMC Pediatr. 2024 Mar 7;23(Suppl 2):655. doi: 10.1186/s12887-023-04495-z (PMC10921557; doi:10.1186/s12887-023-04495-z)
Supplement: Supplementary file 1 — Additional file 1. Evidence-based framework for co-design of mHealth tools and application to the NEST360 design process. [file 12887_2023_4495_MOESM1_ESM.pdf]

# SUPPLEMENTAL INFORMATION – ADDITIONAL FILE 1

## SUPPLEMENT TITLE

**Small and sick newborn care: learning for implementation across Africa and beyond.**

## PAPER TITLE

**Health facility assessment of small and sick newborn care in low- and middle-income countries: systematic tool development and operationalisation with NEST360 and UNICEF**

Additional File 1: *Evidence-based framework for co-design of mHealth tools and application to NEST360 design process.*

| Steps for co design in mHealth tools | Adapted steps used in NEST360 design of data tools |
|---|---|
| Step 1: User needs | 1. Review other tools and end user needs |
| Step 2: Stakeholder needs | |
| Step 3: The thing | 2. Co-design the tool |
| Step 4: Iterate | 3. Operationalise and continue to improve the tool |
| Step 5: Deliver | |
