# Supplementary material for: Health facility assessment of small and sick newborn care in low- and middle-income countries: systematic tool development and operationalisation with NEST360 and UNICEF
Source: BMC Pediatr. 2024 Mar 7;23(Suppl 2):655. doi: 10.1186/s12887-023-04495-z (PMC10921557; doi:10.1186/s12887-023-04495-z)
Supplement: Supplementary file 2 — Additional file 2. List of 866 key health system ingredients for SSNC. [file 12887_2023_4495_MOESM2_ESM.pdf]

SUPPLEMENTAL INFORMATION – ADDITIONAL FILE 2

SUPPLEMENT TITLE

Small and sick newborn care: learning for implementation across Africa and beyond.

PAPER TITLE

Health facility assessment of small and sick newborn care in low- and middle-income countries: systematic tool development and operationalisation with NEST360 and UNICEF

Additional File 2: *List of 866 key health system ingredients for SSNC.*

**Labor and Delivery Room**
**General newborn equipment**
**Infrastructure**
Access to drinking water (for staff and patients)
Access to first stage labour room and postnatal room

Area for newborn resuscitation

Clean storage space for supplies (linen and general equipment)

Communication infrastructure (telephone, internet or radio access (may be shared with other units))

Consistent, uninterrupted 24-hour stabilised power supply

Dedicated space for labour and delivery only

Fan or air conditioning

Health management information system (shared with other units)

Heating arrangements

Lighting to ensure good illumination day and night

Consistent oxygen source/supply

Midwives station/charting or staff work area

Outlets sufficiently available and easily accessible (power strips)

Outlets rated amperage

Sufficient infrastructure for privacy for mothers, including curtains

Toilet or latrine for patients and visitors

Toilet or latrine for staff

Water supply (uninterrupted) (for hand washing, cleaning etc.)

Waiting area for visitors/family

24-hour service availability

Equipment

Back-up generator 25-50 KVA capacity (may be shared with other units)

Blood pressure apparatus (sphygmomanometer)

Blood pressure accessories including neonatal sized blood pressure cuff (sizes 1-5)

Blood sugar glucose dipsticks

Bowls (preferably polypropylene)

Computer (may be shared with other units)

Cot/bassinet/bayonets on castors

Delivery beds (for mothers)

Delivery packs
Doppler
Dressing trolley (or equivalent)
Dressing trays/procedure trays (or equivalent) (sterile and non-sterile)
Emergency trolley (or equivalent)
Endotracheal tubes
Episiotomy scissors
Foetal stethoscope
Flashlight/torch with spare batteries
Forceps (large and medium)
Gauze
Gauze bandages
Glucometer with test strips
Heel lancet
Infusion stands
Infusion kits
IV cannula 22G, 24G, (25G, 28G rarely used)
Kidney bowls (polypropylene, stainless steel) 825ml
Magill's forceps
Manual vacuum extractor
Measuring tape (preferably vinyl coated)
Mucus extractor
Nasogastric feeding tubes 3.5-10 with caps
Oxygen tubing
Paediatric infusion set (60 drops per ml burette)
Partograph
Recharger for batteries
Refrigerator (for drugs etc.)

Scissors

Speculum

Sterile gauze

Sterile needles (19-26 gauge) or butterfly set (23-25 gauge)

Sterile syringes (small sizes 0.5, 1ml, 2ml, 5ml, 10ml, 20ml)

Sterile tissue forceps

Sterile drapes

Stethoscope (neonatal)

Suction pump (portable, electrical with accessories)

Suction pump (manual, non-electric power dependent)

Suture set (needle and materials)

Swabs and/or cotton wool balls

Vital sign monitors (NIBP, HR, SpO2, ECG, RR, Temp) with accessories

Wall clock/timer with second hand

Weighing scales for newborns (preferably with 5-10g increments)

Guidelines, Checklists, Registers

CEmOC and BEmOC guidelines

Classification of breathing difficulty and SpO2 thresholds

Management of pregnancy and childbirth (Integrated management of pregnancy and Childbirth - IMPAC)

Management of pregnancy and newborn complications

National immunisation schedule

Referral and transfer to neonatal unit

Use of radiant warmers

Drug inventory

APGAR

Birth certification papers

Newborn screen filter cards (per national protocol)

General birth records/notes (ongoing medical care)

Health insurance form (if relevant)

Immunisation cards/weight cards

ID labels

Laboratory request forms

Referral forms (if needed)

Birth registration (or access to)

Cause of death certificates

Death/mortuary register

General register for labour and delivery ward

Newborn death stillbirth audit

Referral register

Postpartum ward register

Infection Prevention and Control

Infrastructure

Access to laundry facilities

Areas for hand washing

Area for cleaning and disinfecting supplies and equipment

Autoclave room or sterilization space (can be shared with other units)

Hand washing stations/sinks

Incinerator (e.g. burn in incinerator, off-site incineration can be shared with other units)

Sterilisation space (e.g. space for dry heat, steam, electric boiler or steamer or non-electrics with cover for boiling and steaming & drum and stand) (can be shared with other units)

Storage space for soiled utility

Ventilation

Equipment

Alcohol-based hand rub

Antiseptics (e.g. chlorhexidine 7%, ethanol, povidone-iodine, chlorhexidine gluconate 4% gel, aqueous chlorhexidine 0.05% and 0.2%).

Autoclave (or equivalent sterilisation equipment can be shared with other units)

Contaminated waste bin (leak proof)

Decontamination container

Disinfectant solutions (e.g. chlorine bleach)

Eye shields

Gloves (disposable)

Gloves (sterile)

Gloves (elbow length heavy duty)

Gloves (heavy duty rubber or latex utility gloves)

Hand drying options (paper towels or appropriate hand dryers)

Iodine

Instrument sterilizer

Mayo stand (or equivalent) on castors

Nail brushes or sticks

Non-sterile protective clothing (e.g. plastic aprons)

Pictorial hand washing instructions

Regular trash/waste bin

Receptacle for soiled linen

Sharps containers (puncture proof)

Sluice

Soap for handwashing

Surgical masks, drapes and cap and boots for procedures

Umbilical vein catheters sizes 3.5 and 5 (where expertise available)

HR Skills

Staff trained on interventions

Measurement of regular performance of intervention

Guidelines, Checklists, Registers

Cleaning of instruments and equipment

Cleaning schedule and/or daily cleaning checklist

Infection prevention and control guidelines for the labour and delivery unit and standard precautions

Essential Newborn Care

Infrastructure

As per general and infection control infrastructure for labour and delivery room

Equipment

As per general equipment and infection control items for labour and delivery room

Clean blankets, towels and linen (for drying baby)

Disposable diapers/nappies

Newborn hats/caps (including preterm sizes)

Newborn mittens, socks

Sterile scissors and/or sterile blade to cut cord

Umbilical cord clamp (sterile ligatures or clamp of Barr) or cord ties/sterile thread

Drugs

Vitamin K1 (Phytomenadione) (IM or IV)

Acyclovir 3% topical eye ointment

HR Skills

Staff trained on interventions

Measurement of regular performance of intervention

Guidelines, Checklists, Registers

Essential newborn care guidelines

Thermal protection, including bathing

Immediate and Exclusive Breastmilk Feeding

Infrastructure

As per general and infection control infrastructure for labour and delivery room

Expression space/expressing room and storage for expressed breastmilk (see also sick newborn space)

Space/allowance for patient privacy for mothers

Equipment

As per general equipment and infection control items for labour and delivery room

Educational information on breastfeeding for mothers (e.g. written and pictorial information, support classes or groups, posters)

HR Skills

Staff trained on interventions

Measurement of regular performance of intervention

Guidelines, Checklists, Registers

Standards on immediate and exclusive breastfeeding

Neonatal Resuscitation

Infrastructure

As per general and infection control infrastructure for labour and delivery room and essential newborn care

Newborn emergency space for resuscitaire/newborn resuscitation

Equipment

As per general equipment and infection control items for labour and delivery room and essential newborn care

Airway suction apparatus (suction bulb manual, mechanical or electrical)

Bag self-inflating (neonatal size, ideally with filter)

Neonatal sized face masks (size 0-1)

Nasal prongs 1mm and 2mm (if nasal prongs not available use nasal catheter (8-F and 6-F sizes)

Neonatal sized pulse oximetry probes/sensors for oxygen saturations

Mucus extractor

Oxygen humidifiers

Oxygen low flow device

Oxygen flow splitter for newborn

Oxygen tubing

Resuscitation mannequin (for practice)

Resuscitaire (with heat source)

Suction catheters size 5, 8, 10, 12 & 14 (sterile disposable)

Pulse oximeter

T-piece resuscitator (only where expertise available)

Wall charts/action sequences for neonatal resuscitation (e.g. HBB flowchart)

HR Skills

Staff trained on interventions

Measurement of regular performance of intervention

Guidelines, Checklists, Registers

Newborn resuscitation guidelines

PMTCT

Infrastructure

As per general and infection control infrastructure for labour and delivery room

See laboratory section - access to infrastructure for HIV testing equipment and screening for congenital TB

Equipment

As per general equipment and infection control items for labour and delivery room and essential newborn care

Drugs

Azidothymidine/Zidovudine (AZT) (oral)

Lamivudine

Nevirapine (NVP) (oral)

HR Skills

Staff trained on interventions

Measurement of regular performance of intervention

Guidelines, Checklists, Registers

PMTCT guidelines, including vaccination schedule

PMTCT labour and delivery register

Guidelines protocols on vaccination schedule for HIV exposed infants

Treatment of HIV on the neonatal unit and vaccination schedule for HIV exposed infants

Laboratories and Blood Bank

HIV testing kit

Place for Small and Sick Newborn Care

General for neonatal unit (service availability 24/7)

Infrastructure

24 service availability

Access to drinking water (for staff and patients)

Area for preparing IV drugs and fluids

Clean storage space for supplies (linen, diapers, clinical supplies and general equipment)

Communication infrastructure (telephone, internet or radio access (may be shared with other units))

Consistent, uninterrupted 24-hour stabilised power supply

Dedicated space/room or building (only for small and sick newborns)

Dedicated workspace for doctor on duty

Examination space or area for treatment/stabilisation (allowing for good lighting, warm, with facilities for resuscitation and patient privacy)

Fan or air conditioning with air filters (to prevent risk of airborne infection)

Food provision for mothers that are rooming-in/providing KMC (access to)

Health management information system

Heating arrangements

Lighting to ensure good illumination day and night

Nurses station/charting or staff work area

Outlets sufficiently available and easily accessible (power strips)

Outlets rated amperage

Rooming in facility with chairs and beds for mothers (see kangaroo mother care)

Toilet or latrine for staff

Toilet or latrine for patients and visitors

Water supply (uninterrupted) (for hand washing, cleaning etc.)

Waiting area for visitors/family with educational materials/parent information

Equipment

Adhesive strapping for peripheral lines (or IV film dressing)

Baby nappies/diapers napkins

Back-up generator 25-50 KVA capacity

Blood collection tubes (appropriate small size) e.g. vacuum tubes serum and EDTA 3ml and 6ml

Blood collection tube holders

Blood collection vacuum tube needles 22G
Blood pressure apparatus (sphygmomanometer)
Blood pressure accessories including neonatal sized blood pressure cuff (sizes 1-5)
Blood pressure transducer (for central arterial lines)
Bowls (polypropylene)
IV cannula 22G, 24G, 25G 28G
Capillary sample tubes (glass collection tubes)
Comfortable chairs for mothers
Comfortable chairs for staff
Communication equipment (e.g. radio, telephone)
Computer (for electronic records or data system may be shared with other unit)
Clean blankets, towels and linen including survival blankets
Cots/bayonets for newborns
Cot and incubator mattresses
Dressing trolley (or equivalent)
Dressing trays/procedure trays
Emergency trolley (or equivalent)
Examination lights mobile - 220-12V
Flashlight/torch with spare batteries
Gauze, swabs, cotton wool
Gauze bandages
Heated mattresses (e.g. for hot cots)
Identification bands
Incubators
Infantometer, plexi, 105cm
Intra-osseous needle (or 22G needles)
Kidney bowls (polypropylene, stainless steel) 825ml
Long line packs (for percutaneously inserted central lines)

Lumbar puncture needles (or 23G needles)
Measuring tape (preferably vinyl coated)
Mucus extractor
Newborn hats/caps (including preterm sizes)
Newborn mittens, socks
Padded boards and/or splints for neonates and preterm
Room thermometer
Radiant warmer, fixed height, with trolley, drawers and O2 bottles
Radiant warmer insulating stickers
Radiant warmer probes
Recharger for batteries
Refrigerator (for drugs etc.)
Sample collection tubes (pus, cerebrospinal fluid)
Scissors
Soft gauze tourniquet (or rubber band for scalp vein)
Spacer
Spatula
Sterile equipment stand/dressing tray
Sterile gauze
Sterile tissue forceps
Sterile (low flow) lancet for heel pricks
Sterile blades/scissors
Stethoscope (neonatal)
Suction catheters size 5, 8, 10, 12 & 14 (sterile disposable)
Suction pump (portable, electrical with accessories)
Suction pump (manual, non-electric power dependent)
Suture set
Thermometers (preferable digital for newborns that measure 32°C-43°C - must measure below 35.5°C degrees)

Umbilical vein catheters sizes 3.5 and 5 (where expertise available)

Urinary catheter sizes 5-8

Urine bags (paediatric)

Urine dipsticks (Multistix)

Vital sign monitors (NIBP, HR, SpO2, ECG, RR, Temp) with accessories

Warming crib

Wall clock/timer with second hand

Weighing scales for newborns (preferably with 5-10g increments)

X-ray system (preferably mobile for chest x-ray e.g. for pneumothorax) (may be shared with other units)

X-ray viewer (negatoscope) (may be shared with other units)

CT scanner (may be shared with other units)

Echocardiography

Electrocardiogram (ECG) recorder, portable, with accessories (may be shared with other units)

MRI (unlikely to be available in most settings. If available, may be shared with other units)

Ultrasound scanner (portable) with appropriate probes (e.g. for cranial ultrasound) (not available in all settings and may be shared with other units)

Guidelines, Checklists, Registers

Admission guidelines

Discharge and follow up

Disease specific treatment guidelines (e.g. malaria, tetanus, hepatitis, Zika)

Gestational age assessment

IMNCI

Inborn and outborn infants

Management of gastric residuals

Neonatal practical procedure guideline or advanced neonatal guidelines

National immunisation schedule

Parental/family visitation and access

Referral

Use of incubators and radiant warmers

Visitation
Discharge checklist
Drug inventory
Emergency trolley checklist
Follow up
Prescription charts
Discharge forms
General clinical records/notes (ongoing medical care)
Growth chart (weight, length, head circumference) (premature and infant charts)
Health insurance form (if relevant)
ID labels for inpatients
Lab request forms
Observations charts for recording of vital signs
Referral forms (if needed)
Tetanus observation charts
Birth registration
Cause of death certificates
Civil Vital registration system
Death/mortuary register
Discharge register
Newborn care unit register
Newborn death stillbirth audit
Referral register
Vaccination record/register
**Infection Prevention and Control for the Neonatal Unit**
**Infrastructure**
Access to laundry facilities
Areas for hand washing

Area for cleaning and disinfecting supplies and equipment

Autoclave room or sterilization space (may be shared with other units)

Hand washing stations/sinks

Hand drying towels (single use)

Incinerator (e.g. burn in incinerator, off-site incineration) (may be shared with other units)

Sterilisation space (e.g. space for dry heat, steam, electric boiler or steamer or non-electrics with cover for boiling and steaming & drum and stand) (can be shared with other units)

Storage space for soiled utility

Ventilation

Equipment

Alcohol-based hand rub

Antiseptics (e.g. chlorhexidine 7%, ethanol, povidone-iodine, chlorhexidine gluconate 4% gel, aqueous chlorhexidine 0.05% and 0.2%).

Autoclave (or equivalent sterilisation equipment) (may be shared with other units)

Contaminated waste bin (leak proof)

Decontamination container

Disinfectant solutions (e.g. chlorine bleach)

Gloves (sterile)

Gloves (regular/disposable)

Gloves (heavy duty rubber or latex utility)

Instrument sterilizer

Mayo stand (or equivalent) on casters

Nail brushes or sticks

Non-sterile protective clothing (e.g. plastic aprons)

Pictorial hand washing instructions

Regular trash/waste bin

Receptacle for soiled linen and diapers

Sharps containers (puncture proof)

Sluice (may be shared with other units)

Soap for handwashing

Surgical masks, drapes, gowns and cap for procedures

HR Skills

Staff trained on interventions

Measurement of regular performance of intervention

Guidelines, Checklists, Registers

Cleaning of instruments and equipment

Infection prevention and control for the neonatal unit

Cleaning schedule and/or daily cleaning checklist

Neonatal Resuscitation on neonatal unit

Infrastructure

As per general and infection control infrastructure for small and sick newborn space

See oxygen administration for other items

Equipment

As per general and infection control equipment for small and sick newborn space

Bag and mask self-inflating (neonatal size, ideally with filter)

Oropharyngeal airway/guedel airway

Neonatal sized face masks (size 0-1)

See oxygen administration for other items

HR Skills

Staff trained on interventions

Measurement of regular performance of intervention

Guidelines, Checklists, Registers

Newborn resuscitation guidelines

Kangaroo Mother Care (KMC) including follow up

Infrastructure

As per general and infection control infrastructure for small and sick newborn space

Dedicated, separate room or space for mothers to room in (KMC room/ward)

Private washing areas and toilet for mothers

Food provision for mothers/area for preparation of food

Sufficient space for mothers to store personal items, comfortable chairs and privacy

Equipment

As per general and infection control equipment for small and sick newborn space

Beds for mothers (lateral position) with curtains for privacy

Cabinets for mothers

Caps/hats for small babies

Comfortable chairs for mothers

Insecticide treated bednets for KMC mothers (in malaria endemic areas)

Drugs

See drugs list for supplements, vitamins etc.

HR Skills

Staff trained on interventions

Measurement of regular performance of intervention

Guidelines, Checklists, Registers

Kangaroo mother care

Kangaroo mother care register

Alternative feeding if baby unable to breastfeeding (cup & nasogastric feeding)

Infrastructure

As per general and infection control infrastructure for small and sick newborn space

Area or room for breastmilk expression

Milk room/area for preparing milk feeds and storage of expressed breastmilk

Equipment

As per general and infection control equipment for small and sick newborn space

Adhesive tape/strapping for NG tubes

Bottles, teats, dummies (as appropriate for feeding guidelines)

Breast pumps (battery powered)

Breastmilk substitute (only for babies with mothers unable to express milk)

Collection containers (for expressed breastmilk)

Feeding cups and spoons/paladai/feeding syringes

Litmus paper/testing strips (or equivalent)

Nasogastric feeding tubes 3.5-10 with caps

Educational information on breastfeeding for mothers (e.g. written and pictorial information, posters)

Refrigerator and freezer (for milk storage only)

Sterile feeding syringes (2.5ml, 5ml, 10ml)

Stethoscope

Utensils and containers for preparing milk feeds especially graduated measuring jug/cup

Drugs

See drugs list for supplements, vitamins etc.

HR Skills

Staff trained on interventions

Measurement of regular performance of intervention

Guidelines, Checklists, Registers

Infant feeding for the neonatal unit, including enteral feeding volumes by weight and age, IV fluid volumes

Fluid volumes and medications

Fluid balance and feeding chart (fluid input and output)

Safe administration of oxygen (including equipment for resuscitation)

Infrastructure

As per general and infection control infrastructure for small and sick newborn space

Consistent oxygen source/supply (e.g. oxygen concentrators)

Equipment

As per general and infection control equipment for small and sick newborn space

Apnoea monitor

Bag and mask self-inflating (neonatal size, ideally with filter)

Filter (spare)

Head box (optional)

Mucus extractor

Nasal prongs 1mm and 2mm (if nasal prongs not available use nasal catheter (8-F and 6-F sizes)

Neonatal sized face masks (size 0-1)

Neonatal sized pulse oximetry probes/sensors

Oxygen blenders

Oxygen humidifiers

Oxygen low flow device

Oxygen flow splitter for newborn

Oxygen meter

Oxygen tubing

Pulse oximeters (bedside)

Resuscitation mannequin (for training and practice)

Suction bulbs

Suction catheters size 5, 8, 10, 12 & 14ch

Suction pump (portable, electrical with accessories)

Suction pump (manual, non-electric power dependent)

Drugs

See drugs list

HR Skills

Staff trained on interventions

Measurement of regular performance of intervention

Guidelines, Checklists, Registers

Classification of breathing difficulty and SpO2 thresholds and protocols for oxygen therapy and monitoring

Intravenous fluids and management of hypoglycaemia

Infrastructure

As per general and infection control infrastructure for small and sick newborn space

See laboratory section

Separate area/clean space for preparing IV fluids (can be the same area as for preparation of IV drugs)

Equipment

As per general and infection control equipment for small and sick newborn space

Adhesive tape for syringe pump

Butterfly sets (22-25 gauge)

Glucometer

IV tubing/infusion set (neonatal giving set) with burette 100-150ml, sterile, single use

IV infusion stands on castors

Sterile needles (19-26 gauge) or butterfly set (23-25 gauge)

Sterile syringes (small sizes 0.5, 1ml, 2ml, 5ml, 10ml, 20ml)

Stopcocks 2 or 3 way

Syringe driver/syringe pumps 10, 20, 50ml (single phase)

Rapid blood sugar testing strips/paper reagent strips or equivalent

Drugs

Calcium gluconate 10%

Dextrose 10% with normal saline

Dextrose/glucose 5%

Dextrose/glucose 10%

Potassium chloride (KCL) 7.5%, 10%, 15%

Sodium bicarbonate

Sodium chloride 0.9%

Ringer’s lactate

Water for injection

HR Skills

Staff trained on interventions

Measurement of regular performance of intervention

Guidelines, Checklists, Registers

Fluid volumes and medications

Injectable antibiotics for neonatal infection

Infrastructure

As per general and infection control infrastructure for small and sick newborn space

Lab infrastructure for septic screening (blood culture, Full blood count, C-Reactive Protein) (see lab)

Separate area/clean space for preparing IV drugs (can be the same as area for IV fluids)

Equipment

As per general and infection control infrastructure for small and sick newborn space

See general equipment and specific equipment for IV fluids

Drugs

Amoxicillin (oral suspension)

Amoxicillin (injection)

Amikacin

Ampicillin (IV or IM)

Ampicillin (oral)

Azithromycin (oral)

Benzathine benzylpenicillin (IM)

Benzylpenicillin (Penicillin G) (IV or IM)

Cefalexin (oral suspension)

Cefotaxime (IV or IM)

Ceftriaxone (IV or IM)

Ciprofloxacin (injection)

Ciprofloxacin (oral)

Clindamycin (IV)

Co-amoxiclav (oral suspension)

Co-amoxiclav (injection)

Cotrimoxazole (oral)

Erythromycin (oral)

Flucloxacillin (IV/IM) (cloxacillin)

Flucloxacillin (oral)

Gentamicin (IM or IV)

Isoniazid (oral)

Kanamycin

Metronidazole (IV)

Metronidazole (oral)

Penicillin G Procaine (IM only)

Procaine benzylpenicillin (IM)

HR Skills

Staff trained on interventions

Measurement of regular performance of intervention

Guidelines, Checklists, Registers

Drug doses, dilutions and preparations for the neonatal unit

Medication formulary (with neonatal doses) or prescribing guideline

Prescription chart

Effective Phototherapy

Infrastructure

As per general and infection control infrastructure for small and sick newborn space

See laboratory section for bilirubin levels (or transcutaneous bilirubinometer on ward)

Equipment

As per general and infection control equipment for small and sick newborn space

Calibration materials

Cuvettes

Eye patches/eye shields for baby

Exchange transfusion sets

Icterometer

Irradiance meter/spectro-radiometer

Phototherapy lamps/units with fluorescent tubes (high intensity) or LED phototherapy

Spare fluorescent tubes/LEDs

Test strips

Bilirubinometer

White linen for babies on phototherapy for cot and to cover unit

HR Skills

Staff trained on interventions

Measurement of regular performance of intervention

Guidelines, Checklists, Registers

Treatment thresholds for phototherapy and exchange transfusion

Use of phototherapy units (e.g. positioning of phototherapy lamps, baby etc.)

Seizure Management

Infrastructure

As per general and infection control infrastructure for small and sick newborn space

See laboratory section

Equipment

As per general and infection control equipment for small and sick newborn space

Drugs

Diazepam (oral/NG)

Diazepam emulsion (IV)

Midazolam (oral solution)

Paraldehyde (rectal)

Phenobarbital (IV or IM)

Phenobarbital (oral)

Phenytoin (IV)

HR Skills

Staff trained on interventions

Measurement of regular performance of intervention

Guidelines, Checklists, Registers

Management of newborn convulsions and spasms

Continuous Positive Airway Pressure and Assisted/Mechanical Breathing

Infrastructure

As per general and infection control infrastructure for small and sick newborn space

See laboratory section

Equipment

As per general and infection control equipment and safe oxygen therapy equipment for small and sick newborn space

CO_2 detector

Chest drain set

CPAP driver system (standard or bubble CPAP) with accessories (may vary dependent on CPAP system used)

CPAP tubing

Distilled water

Drainage tubing and under water seal drainage bottle plus accessories

Endotracheal tubes (disposable cuffed or uncuffed), sizes 2.0, 2.5, 3.0 and 3.5

Endotracheal tube introducers

Infant laryngoscope set with spare bulb and batteries

Infant laryngoscope (0,1 blades)

Large forceps (e.g. spencer wells)

Laryngoscope light bulb (spare)

Oxygen air blenders

Pump suction, portable, bottle with accessories

Prongs

Nasogastric suction tubes 3.5, 5, 8, 10

Respirator/ventilators plus accessories

Portable patient monitors

ECG leads

Temperature probes

Pulse oximetry probes

ECG stickers

Insulating stickers (temperature)

Transilluminators

Drugs

See drugs section

HR Skills

Staff trained on interventions

Measurement of regular performance of intervention

Blood Transfusion for Newborns

Infrastructure

Blood bank (see lab and blood bank for specifics)

Equipment

Neonatal blood transfusion set

4-way stopcock for umbilical venous line

Exchange transfusion sets

Portable monitors

Drugs

See essential drugs list

HR Skills

Staff trained on interventions

Measurement of regular performance of intervention

Guidelines, Checklists, Registers

Blood transfusion and exchange transfusion procedural guidelines

Transfusion surveillance/vital signs document (for blood transfusions)

Reporting forms for adverse events (including blood transfusion reaction reporting)

Treatment and screening for retinopathy of prematurity

Infrastructure

As per general and infection control infrastructure for small and sick newborn space

Ophthalmology service (does not need to be 24 hour)

Equipment

As per general and infection control equipment and safe oxygen therapy equipment for small and sick newborn space

Indirect ophthalmoscope (with small pupil adjustments) x1 per neonatal unit

Condensing lenses 20D and 28D for indirect ophthalmoscope

Neonatal lid speculums (Alfonso)

Scleral depressors (Schoket/wire vectis)

Solution for corneal wetting (e.g. Ringer's lactate)

Newborn pulse oximeter

Laser (can be shared between more than one facility/service)

Portable diode/green laser with indirect delivery system (can be shared between more than one facility)

Laser goggles

Drugs

Dilating eye drops (tropicamide 0.5% + phenylephrine 2.5%)

Local anaesthetic eye drops (proparacaine 0.5%)

Artificial tear drops - for lubrication during procedure

Antibiotic drops (moxifloxacin/betadine) - at end of procedure

HR Skills

Staff trained on interventions

Measurement of regular performance of intervention

Guidelines, Checklists, Registers

Classification of breathing difficulty and SpO_2 thresholds and protocols for oxygen therapy and monitoring

Referral Service

Infrastructure

Vehicle maintenance infrastructure (or access to)

Communication for transport vehicle (e.g. radio)

Fuel source/system

Equipment

Adhesive tape
Antiseptic solution
Blankets/linens
Butterfly set or cannula
Cotton wool balls and /or gauze
Diapers/napkins
Fuel for transport vehicle
Gastric tubes (size 5, 6, 7, 8)
Gloves
Hats, socks, mittens (for baby)
IV infusion set
Resuscitation bag and mask
Portable suction apparatus
Pulse oximetry
Oxygen cylinder with flow meter
Oxygen cylinder (portable)
Nasal prongs/and or nasal catheter
Source of warmth
Stethoscope
Sterile needles (19-26 gauge) or butterfly set (23-25 gauge)
Sterile syringes (small sizes 0.5, 1ml, 2ml, 5ml, 10ml, 20ml)
Torch with extra batteries and bulb
Thermometer
Transport incubator(s)
Transport vehicle (type may vary by context)
Wraps or cloths for kangaroo position

Drugs

IV fluids

Drugs/medicine (any medicine newborn is taking if receiving a dose during trip)

Expressed breastmilk (if baby is able to feed or, preferably, mother with newborn)

Documentation

Referral guidelines

Distance to nearest intensive care and special care unit and contact number of centre with advanced care

Detailed documents with patient notes (drugs and other treatment given before transport)

Human Resources

Provider

Administration staff

Anesthetist

Biomedical engineer

Cleaners

Community health worker (or equivalent)

Driver (transport vehicle)

General medical doctor

Health officer/Clinical officer (may be context specific)

Health information technologist/data manager

Lab scientist

Lab technician

Lactation counsellor/infant feeding coordinator

Midwifery professional (e.g. enrolled midwife, degree, diploma)

Nursing professional

Neonatal nurse (or nursing/midwifery professionals with specialist training in sick newborn care)

Neonatologist

Nutritionist

Obs/gyne doctor

Other nursing professionals (e.g. enrolled nurse), nursing assistants or auxiliaries

Other anesthetics staff (e.g. nurse anesthetist, diploma etc.)

Ophthalmologist

Pathologist

Pediatrician

Pharmacist

Pharmacy technicians/assistants

Porters

Psychologist

Physiotherapist

Radiographer

Security staff

Social worker

Speech therapist (or equivalent)

Surgeon

Medicines/Drugs

Pharmacy infrastructure

Pharmacy service availability 24/7

Supply management system

Refrigeration for vaccination

Refrigeration for other drugs/medicines

Safe drug storage conditions (protection from moisture, heat, infestation)

Drug inventory (also in health information section)

Antiretrovirals (may vary depending on national HIV guidelines)

Azidothymidine/Zidovudine (AZT) (oral)

Lamivudine

Nevirapine (NVP) (oral)

Anticonvulsants

Diazepam (oral/NG)

Diazepam emulsion (IV)

Midazolam (oral solution)

Paraldehyde (rectal)

Phenobarbital (IV or IM)

Phenobarbital (oral)

Phenytoin (IV)

Analgesics

Ibuprofen (IV)

Morphine (IV)

Morphine (oral)

Paracetamol (oral)

Paracetamol (suppository)

Paracetamol (injection)

Anti-malarials

Artesunate (IV or IM)

Artesunate (rectal)

Arthemeter (IM)

Artemisinin-based combined therapy (oral)

Antibiotics

Amoxicillin (oral suspension)

Amoxicillin (injection)

Amikacin

Ampicillin (IV or IM)

Ampicillin (oral)

Azithromycin (oral)

Benzathine benzylpenicillin (IM)

Benzylpenicillin (Penicillin G) (IV or IM)

Cefalexin (oral suspension)

Cefotaxime (IV or IM)

Ceftriaxone (IV or IM)

Ciprofloxacin (injection)

Ciprofloxacin (oral)

Clindamycin (IV)

Co-amoxiclav (oral suspension)

Co-amoxiclav (injection)

Cotrimoxazole (oral)

Erythromycin (oral)

Flucloxacillin (IV/IM) (cloxacillin)

Flucloxacillin (oral)

Gentamicin (IM or IV)

Isoniazid (oral)

Kanamycin

Metronidazole (IV)

Metronidazole (oral)

Penicillin G Procaine (IM only)

Procaine benzylpenicillin (IM)

Tetracycline 1% eye ointment

Antibiotic drops (moxifloxacin/betadine) - at end of procedure

Corticosteroids

Betamethasone (IM)*

Dexamethasone (IM) *

Emergency Drugs

Adrenaline/epinephrine (IV)

Aminophylline

Atropine (injection)

Calcium gluconate 10% (injection)

Hydrocortisone (injection)

Magnesium sulphate (IV)

Naloxone (IV)

IV fluids

Calcium gluconate 10%

Dextrose 10% with normal saline

Dextrose/glucose 5%

Dextrose/glucose 10%

Potassium chloride (KCL) 7.5%, 10%, 15%

Sodium bicarbonate

Sodium chloride 0.9%

Ringer's lactate

Water for injection

Vaccines

BCG vaccine

Diphtheria

Pertussis vaccine

Haemophilus influenza type b (Hib) vaccine

Hepatitis B vaccine

Tetanus toxoid

Oral poliomyelitis vaccine

Other Drugs

Aciclovir (IV)

Acyclovir 3% topical eye ointment

Anti-Rho (D) immune globulin (injection)

Caffeine citrate (oral)

Caffeine citrate (IV)

Chlorhexidine digluconate 7.1% gel (delivering 4% chlorhexidine)

Domperidone

Ethambutol
Ferrous fumerate (oral syrup)
Folic acid
Fluconazole (IV)
Fluconazole (oral)
Furosemide (IV)
Furosemide (oral)
Glycerin chip
Hepatitis B immune globulin (HBIG)
Human milk fortifier
Insecticide treated bed nets (in malaria endemic areas)
Lidocaine solution
Miconazole cream (or equivalent e.g. gentian violet)
Multivitamin
Nystatin (oral solution)
Nystatin cream
Omeprazole (IV)
Omeprazole (oral)
Oral rehydration solution
Oxygen supply
Phosphate and calcium supplements
Potassium chloride (1mmol/ml) (oral)
Pyridoxine (oral)
Ranitidine (IV)
Ranitidine (oral)
Rifampicin
Pyrazinamide
Sucrose 30% (oral)

Surfactant (not likely to be available in many settings due to cost)

Tetanus immunoglobulin (HTIG) (IM)

Vitamin B6 (pyridoxine) (IV or IM)

Vitamin D

Vitamin K1 (Phytomenadione) (IM or IV)

Water based lubricant

Dilating eye drops (tropicamide 0.5% + phenylepherine 2.5%)

Local aneasthetic eye drops (proparacaine 0.5%)

Artificial tear drops - for lubrication during procedure

Zinc oxide cream

Laboratories and Blood Bank

Biochemistry should be able to perform:

Blood glucose (e.g. glucometer and test strips)

C-reactive protein (CRP)

Electrolytes (urea, sodium, potassium, calcium, magnesium and creatinine)

Serum bilirubin (or transcutaneous bilirubinometers as cheaper alternative)

Cerebrospinal fluid analysis (glucose and protein)

Liver function testing

Blood gas analysis

Urine dipstick for urinalysis that measure: pH, proteins, glucose, ketones, blood nitrates, leucocytes

Glucose 6-phosphate deyrogenase (G6PD) screening

Haemotology should be able to perform:

Blood typing and cross matching

Coagulation profile

Coombs test

Full blood count (FBC)/Full blood examination (FBE)

Haemaglobin (e.g. haemacue, haemoglobin colour scale refill kit/starter kit) and or haematocrit testing (erythrocyte volume fraction)

Microbiology should be able to perform:

Culture and sensitivity on samples of blood, pus, cerebrospinal fluid and urine

Cerebrospinal cell count

Gram staining

Stool analysis

STI Testing including

Chlamydia testing

Enzyme immuno assay (EIA), gonorrhea Ag, kit

Hepatitis B surface antigen (HbsAg)

HIV testing kit

Syphilis testing

Other important lab tests:

TB testing (tuberculin skin testing, access to Ziehl-Neilsen staining and ideally GeneXpert)

Malaria testing (preferably blood film microscopy as more reliable option than rapid diagnostic tests for neonatal malaria)

Glucose 6-phosphate deyrogenase (G6PD) screening

Thyroid function tests

Blood bank should have facilities to perform:

Blood typing and cross matching

Coombs test

Storage of fresh whole blood, including type 0, Rh-negative blood, packed cells and fresh frozen plasma

Blood screening for HIV, Hep B, Hep C, Syphilis and malaria

Blood transfusion guidelines for neonates

Documentation

Documentation for newborn unit (labour and delivery)

GUIDELINES AND/OR PROTOCOLS for the following:

CEmOC and BEmOC guidelines

Classification of breathing difficulty and SpO2 thresholds

Cleaning of instruments and equipment

Essential newborn care guidelines

Infection prevention and control guidelines for the labour and delivery unit and standard precautions

Management of pregnancy and childbirth (Integrated management of pregnancy and Childbirth - IMPAC)

Management of pregnancy and newborn complications

Newborn resuscitation guidelines

National immunisation schedule

PMTCT guidelines, including vaccination schedule

Referral and transfer to neonatal unit

Standards on immediate and exclusive breastfeeding

Use of radiant warmers

CHECKLISTS

Cleaning schedule and/or daily cleaning checklist

Drug inventory

FOR EACH NEWBORN

APGAR

Birth certification papers

Newborn screen filter cards (per national protocol)

General birth records/notes (ongoing medical care)

Health insurance form (if relevant)

Immunisation cards/weight cards

ID labels

Laboratory request forms

Referral forms (if needed)

GENERAL REGISTERS/LOG BOOKS

Birth registration (or access to)

Cause of death certificates

Death/mortuary register

General register for labour and delivery ward

Newborn death stillbirth audit

Referral register
Postpartum ward register
PMTCT labour and delivery register
Guidelines protocols on vaccination schedule for HIV exposed infants
**Documentation (place for small and sick newborn)**
