# Supplementary material for: Health facility assessment of small and sick newborn care in low- and middle-income countries: systematic tool development and operationalisation with NEST360 and UNICEF
Source: BMC Pediatr. 2024 Mar 7;23(Suppl 2):655. doi: 10.1186/s12887-023-04495-z (PMC10921557; doi:10.1186/s12887-023-04495-z)
Supplement: Supplementary file 4 — Additional file 4. Recommended equipment for health facility assessments for SSNC. [file 12887_2023_4495_MOESM4_ESM.pdf]

SUPPLEMENTAL INFORMATION – ADDITIONAL FILE 4

SUPPLEMENT TITLE

Small and sick newborn care: learning for implementation across Africa and beyond.

PAPER TITLE

Health facility assessment of small and sick newborn care in low- and middle-income countries: systematic tool development and operationalisation with NEST360 and UNICEF

Additional File 4: *Recommended equipment for Health Facility Assessments*

**Recommended equipment**
1. Tablet with charger and case (1/enumerator)<br>2. Power cord to charge multiple tablets at once<br>3. Mobile Wifi box (1/team)<br>4. Backup paper HFA with clipboard and pen (1/enumerator)<br>5. Hand sanitizer, PPE, disposable shoe covers, head covers (1/enumerator)<br>6. Name tags and bag to carry items (1/enumerator)<br>7. Equipment testers, including Photoradiometer and Oxygen analyzer (1/team)<br>8. Measuring tape, ruler, and spare paper (2/team)<br>9. Pencils, pencil sharpeners, eraser (2/team)<br>10. Copy of hospital introduction letter (1/team)
