# Supplementary material for: Health facility assessment of small and sick newborn care in low- and middle-income countries: systematic tool development and operationalisation with NEST360 and UNICEF
Source: BMC Pediatr. 2024 Mar 7;23(Suppl 2):655. doi: 10.1186/s12887-023-04495-z (PMC10921557; doi:10.1186/s12887-023-04495-z)
Supplement: Supplementary file 5 — Additional file 5. NEST360/UNICEF health facility assessment content summary. [file 12887_2023_4495_MOESM5_ESM.pdf]

SUPPLEMENTAL INFORMATION – ADDITIONAL FILE 5

SUPPLEMENT TITLE

Small and sick newborn care: learning for implementation across Africa and beyond.

PAPER TITLE

Health facility assessment of small and sick newborn care in low- and middle-income countries: systematic tool development and operationalisation with NEST360 and UNICEF

Additional File 5: *NEST360 UNICEF Health Facility Assessment Content Summary*

**HFA modules and components**	**Description**
**1A. Facility Infrastructure**	
Facility Identification	Facility type and level<br>Catchment area
Facility Infrastructure	Facility patient numbers and beds<br>Facility power and backup power sources and functionality<br>Facility water and backup water sources and functionality<br>Facility oxygen systems and fire safety<br>Facility sterile supplies
Physical areas	Physical areas available at the facility<br>Electricity and water availability by facility area

Autoclave and sterilisation	Autoclaving items availability and functionality
Communication infrastructure	Communication method availability and functionality, and use for referral<br>Communication systems reliability and financial policies
Transportation	Transport method availability and functionality<br>Transport maintenance and support systems
**1B. Neonatal Infrastructure**	
Neonatal unit infrastructure	Neonatal unit capacity<br>Neonatal unit transfer infrastructure from labour ward<br>Dedicated areas in neonatal unit<br>Temperature and heating in neonatal unit<br>Neonatal unit lighting<br>Neonatal unit walled/piped oxygen availability<br>Neonatal unit water and backup water sources<br>Fire prevention in neonatal unit
Neonatal unit infection prevention and control	Guideline availability and accessibility<br>Infection surveillance<br>Hand hygiene options and policies on the neonatal unit<br>Toilet/latrine options for neonatal unit staff/visitors<br>Neonatal unit sterilisation and equipment disinfection<br>Waste management on the neonatal unit<br>Staff/visitor personal items and dedicated areas
Neonatal unit electricity	Neonatal unit power and backup power sources and functionality<br>Neonatal unit outlets and power strips<br>Power quality assessment
Neonatal unit layout	Layout diagrams and pictures of each neonatal unit room
Admission and referral criteria	Admission criteria for neonates to the neonatal unit, paediatric ward, and Kangaroo Mother Care (KMC) unit

	Discharge criteria for neonates from the neonatal unit and KMC unit<br>Referral criteria for neonates to lower and higher-level facilities
**2A. Pharmacy and Laboratory**	
Pharmacy	Pharmacy infrastructure and systems<br>Inventory and supply chain processes<br>Medicine availability and stockouts at central pharmacy
Laboratory equipment, supplies, and testing	Laboratory infrastructure, systems, and guidelines<br>Laboratory management meetings<br>Inventory and forecasting of lab supplies<br>Equipment availability and functionality<br>Laboratory supplies availability and stockouts<br>Laboratory tests availability
Laboratory capacity for microbiology	Blood and Cerebrospinal Fluid (CSF) culture methods<br>Blood and Cerebrospinal Fluid (CSF) isolate identification methods<br>Antibiotic sensitivity testing method<br>Laboratory protocols
Laboratory linkage to neonatal unit	Cultures for neonatal unit<br>Communication between laboratory and neonatal unit<br>Laboratory staffing
Blood bank	Blood bank tests availability<br>Blood transfusion availability and supply
Neonatal unit medicines	Medicine availability and stockouts at neonatal unit pharmacy
**2B. Medical Devices and Supplies**	
Infection prevention supplies	Infection prevention supplies availability and stockouts
Inventory and forecasting	Consumable inventory and forecasting
Neonatal care devices and supplies	Device availability and functionality<br>Consumable availability and stockouts
Maintenance and repair	Maintenance support and timeliness of repair

**2C. Biomedical Workshop**	
Spare parts and tools	Workshop personal protective equipment<br>Spare parts availability and stockouts<br>Repair tools availability and functionality
Storage and layout	Infrastructure for storage location of new equipment<br>Workshop space and organisation<br>Layout diagrams and pictures of workshop
Other workshop questions	Workshop documentation<br>Preventive and corrective maintenance processes<br>Decommissioning<br>Equipment leasing and servicing contracts<br>Electronic maintenance system<br>Maintenance reporting<br>Maintenance staffing and scheduling
Maintenance and repair	Maintenance support and barriers to repair<br>Technical staff training
**3. Human Resources**	
Facility and neonatal staffing	Facility and neonatal unit staffing allocation and numbers<br>Neonatal unit shifts
Facility policies and working conditions	Contract and job description<br>Staff rotations<br>Staff cell phone policy
Clinical care guidelines	Guideline availability and accessibility
Newborn care signal functions	Provision of clinical competencies by staff cadre<br>Provision of free newborn care services
Supervisory support and motivation	Clinical staff training<br>Perceived supervisory support<br>Motivation<br>Working conditions
**4. Information Systems**	

Data sources (forms, registers, tally sheets)	Forms, registers, and tally sheets used on the neonatal unit<br><br>Register completion<br><br>Forms and registers supply and stockouts<br><br>Reports generated for the neonatal unit
Filing systems	Filing systems on the neonatal unit<br><br>Storage of medical records for babies discharged alive<br><br>Storage of medical records for deceased babies after discharge<br><br>Storage of neonatal unit admissions register<br><br>Medical record identification system
Neonatal data clerks	Data clerk staffing and responsibilities
Summary data for reporting	Indicators used at facility<br><br>District Health Information Software (DHIS2) staffing and reporting
Maternal perinatal death surveillance and response (MPDSR)	MPDSR tools and dataset
Civil registration and vital statistics (CRVS)	CRVS system and processes
Electronic infrastructure	Electronic information systems availability and management<br><br>Infrastructure for electronic information systems
Indicator variables	Variables collected on each baby admitted to the neonatal unit
**5. Leadership and Governance**	
Target setting	Facility and neonatal unit targets
Financing reports	Financial management policies and reporting
Staff absenteeism & performance review	Staff absenteeism<br><br>Performance review<br><br>Staff training plans
Clinical audit & management meetings	Team meeting records for quality improvement, infection prevention and control, and Maternal

	Perinatal Death Surveillance and Response (MPDSR)
**6. Family-Centred Care**	
Policies and training	Guideline availability and accessibility<br><br>Trainings in Family-Centred Care
Family satisfaction	Mechanisms to voice grievances
Family involvement	Information and involvement with sick baby
Infrastructure	Sitting, sleeping and visitor infrastructure<br><br>Feeding infrastructure<br><br>Dedicated areas for caretakers/families<br><br>Kangaroo Mother Care (KMC) infrastructure and occupancy
**7. Hand Hygiene Observation**	
Hand hygiene behaviour	Observed hand hygiene behaviour on neonatal unit
