# Supplementary material for: Health facility assessment of small and sick newborn care in low- and middle-income countries: systematic tool development and operationalisation with NEST360 and UNICEF
Source: BMC Pediatr. 2024 Mar 7;23(Suppl 2):655. doi: 10.1186/s12887-023-04495-z (PMC10921557; doi:10.1186/s12887-023-04495-z)
Supplement: Supplementary file 6 — Additional file 6. Health facility assessment summary report template. [file 12887_2023_4495_MOESM6_ESM.pdf]

Health Facility Assessment Summary Feedback
Facility:
Assessment Date:

Number of annual births:
Number of annual admissions to neonatal unit:
Facility level:
Approximate population in catchment area:

Infrastructure

Neonatal Unit Capacity		Designated Areas	
Number of cots		Designated area for high risk/acute babies	
Neonatal unit capacity (including cot/radiant warmer/incubator)		Designated area for low risk/stable babies	
Number of babies in neonatal unit on day of HFA visit		Inborn isolation area	
% bed capacity filled		Outborn isolation area	
Frequency of more than 1 baby per cot		Neonatal admissions only to neonatal unit or KMC	
Frequency of more than 1 baby per radiant warmer/incubator		Area for examination and triage when newborn first arrives	
**Electric Power**			
Fuel generator available in facility		Facility connected to electricity grid	
Fuel generator functional		Stable power (no interruption) for last 7 days	
Battery inverter available in facility		Lighting in neonatal unit covered by backup power	
Battery inverter functional		Equipment in neonatal unit covered by backup power	
Solar power available in facility		No equipment damaged from electricity in last year	
Solar power functional			
**Electricity Safety**		**Fire Safety**	
Floor free of cables (cables not running across floor)		Fire extinguisher available in neonatal unit	
Voltage stabilizer on neonatal unit		Date of last inspection	
Power audit certification in last year		Neonatal evacuation plan available	

Respectful & Family Centred Care

Respectful Care		KMC Capacity	
Functioning family toilets available		Number of KMC beds	
Functioning bathing facilities available		Number of KMC beds that sit up	
Mothers/caretakers can visit at any time		Number of KMC reclining chairs	
Caretakers receive formal counselling before discharge		KMC beds meet demand	

N/A = Not applicable   N/R = Not recorded

Health Facility Assessment Summary Feedback
Facility:
Assessment Date:

Medical Devices and Supplies

Device and Consumable Availability						
Digital Scale	Digital Thermometer	Radiant Warmer	Incubator	Phototherapy	Glucometer	Syringe Pump
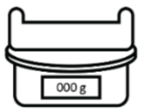	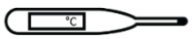	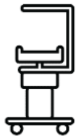	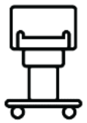	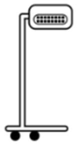	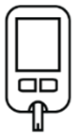	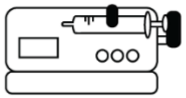
		Probes			*Glucosticks*	
		•			•	

Pulse Oximeter	Suction Pump	Flow Splitter	Bottled Oxygen	Oxygen Concentrator	CPAP
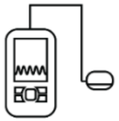	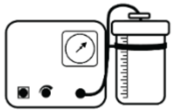	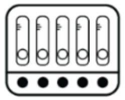	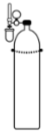	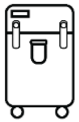	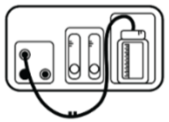
Probes	*Catheters*		*Nasal prongs*		*CPAP prongs*
•	•		•		•
•	•		•		•

Available = available at time of visit, Not available = not available at time of visit, no stockout = no stockout for last 4 weeks

No associated consumables tracked for device

Guidelines	Available	Accessible	Equipment Maintenance	
Thermal protection			Preventive maintenance provided	
Breastfeeding standards			Preventive maintenance system	
KMC practice			Corrective maintenance system	
Oxygen therapy			# Biomed techs/engineers employed by facility	
Fluids, volumes, medications			Biomed techs/engineers employed by facility full-time	
Phototherapy treatment			Biomed present or on-call the night before the HFA visit	

N/A = Not applicable   N/R = Not recorded

Health Facility Assessment Summary Feedback
Facility:
Assessment Date:

Human Resources

Staffing					
**includes pediatricians and neonatologists* *±exclusively assigned*	Nurse±	Clinical Officer	General Doctor	Spec. Doctor*	Data Clerk±
Assigned to neonatal unit					
On duty at time of visit					N/A
On duty the night before					N/A
Ratio of babies / staff during visit					N/A

Information Systems and Governance

Registers and Reporting	
Neonatal unit admissions register used	
Neonatal unit discharge register used	
Electronic summary data submitted (DHIS2)	
**Clinical Audit and Management Meetings**	
Mortality audit/MPDSR team in place	
Mortality audit/MPDSR at least every 3 months	
Quality improvement team in place	
Quality improvement at least every 3 months	

Infection Prevention

Wash			
No regular water shortage in neonatal unit		Waste bin discarded medicines (black)	
Backup water available in neonatal unit		Waste bin infectious (yellow)	
Sinks functioning in neonatal unit		Waste bin anatomical (red)	
Soap or hand sanitizer available in neonatal unit		Sharps container available	
Hand washing wall chart available		Autoclave available	
Hand washing wall chart accessible		Autoclave functioning	
Staff toilets cleaning frequency		Trash bins collected at least once per day	

Laboratory

Laboratory able to perform	
Culture on samples of blood	
Sensitivity on samples of blood	
Culture on samples of CSF	
Sensitivity on samples of CSF	
Serum bilirubin test	
C-reactive protein (CRP)	
Full blood count/examination (FBC/FBE)	

Pharmacy

In stock for last 3 months	
Gentamicin	
Benzylpenicillin	
Amoxicillin	
Ampicillin	
Phenobarbitone	
Caffeine or aminophylline	
Vitamin K	

N/A = Not applicable   N/R = Not recorded
