# Supplementary material for: Health facility assessment of small and sick newborn care in low- and middle-income countries: systematic tool development and operationalisation with NEST360 and UNICEF
Source: BMC Pediatr. 2024 Mar 7;23(Suppl 2):655. doi: 10.1186/s12887-023-04495-z (PMC10921557; doi:10.1186/s12887-023-04495-z)
Supplement: Supplementary file 7 — Additional file 7. Health facility assessment full report template. [file 12887_2023_4495_MOESM7_ESM.pdf]

HEALTH FACILITY ASSESSMENT
FOR SMALL AND SICK NEWBORN CARE
BASELINE COMPLETE REPORT

Date of HFA Visit -

HEALTH FACILITY ASSESSMENT FOR SMALL AND SICK NEWBORN CARE BASELINE COMPLETE REPORT

Table of Contents

List of Acronyms 4

Summary 5
- Facility Overview 5
- Small and Sick Newborn Care Patient Services Provided 5

I. BACKGROUND 6

Purpose 6

Methodology 6
- Tool Development 6
- Training and Data Collection 8
- Data Analysis 9
- References 10

II. HFA BASELINE SUMMARY REPORT 0

III. HEALTH SYSTEMS BUILDING BLOCKS 0

1. INFRASTRUCTURE 0

1.A Facility Infrastructure 0
- 1.A.1 Patient Numbers and Beds 0
- 1.A.2 Power and Water 0
- 1.A.3 Oxygen Systems, Fire Safety, and Other Infrastructure 1
- 1.A.4 Physical Areas Available 1
- 1.A.5 Communication Systems and Infrastructure 2
- 1.A.6 Transportation 3

1.B Neonatal Unit Infrastructure 4
- 1.B.1 Neonatal Unit Capacity and Transfer 4
- 1.B.2 Neonatal Unit Dedicated Spaces 5
- 1.B.3 Neonatal Unit Temperature, Lighting, Oxygen Systems, Water and Fire Prevention 6
- 1.B.4 Neonatal Unit Electricity and Power 7
- 1.B.5 Admission and Referral Criteria 7

2. MEDICAL DEVICES AND SUPPLIES, PHARMACY, AND LABORATORY 10

2.A Medical Devices and Supplies 10
- 2.A.1 Neonatal Equipment & Supplies 10
- 2.A.2 Maintenance and Repair 16

2.B Pharmacy 24
- 2.B.1 Pharmacy Systems 24
- 2.B.2 Essential Drug Availability at Central Pharmacy 25
- 2.B.3 Essential Drug Availability on Neonatal Unit 27

2.C Laboratory 28
- 2.C.1 Laboratory Systems 28
- 2.C.2 Laboratory Equipment and Supplies 28
- 2.C.3 Blood Bank Testing and Supplies 30

3. HUMAN RESOURCES 31
3.A Facility Staffing 31
3.B Policies and Working Conditions 33
3.C Staff Providing Care on the Neonatal Unit 33
3.D Clinical Care Policies and Guidelines 34
3.E Newborn Care Signal Functions 35
3.E.1 Signal Function Performance 35
3.E.2 Staff Who Routinely Provide Each Signal Function 35
3.F Clinical Experience and Training 36
4. INFORMATION SYSTEMS 38
4.A Data Sources 38
4.A.1 Medical Forms 38
4.A.2 Hospital Registers 39
4.A.3 Hospital Tally Sheets 39
4.A.4 Health Summary Reports 40
4.A.5 Forms and Registers Supply 40
4.A.6 Register Completion on Weekends and After Hours 40
4.A.7 Filing systems 40
4.A.8 Storage of medical records 40
4.B Neonatal Data Clerks 41
4.C Summary Data for Reporting 41
4.E Mortality Audit and Maternal Perinatal Death Surveillance and Response 42
4.F Civil Registration and Vital Statistics (CRVS) 43
4.G Electronic Information Systems and Infrastucture 43
4.G.1 Electronic Information Systems 43
4.G.2 Electronic Information Infrastructure 43
5. GOVERNANCE AND LEADERSHIP 44
5.A Target Setting 44
5.B Financing Reports 44
5.C Staff Absenteeism and Performance 44
5.D Inventory and Forecasting of Consumables 44
5.E Hospital Quality Improvement Team 44
5.F Infection Prevention and Control (IPC) Team 45
5.G Maternal and Perinatal Death Surveillance and Response (MPDSR) Team 45
5.H Lab Management and Inventory 45
5.I Newborn Services and Price Details 45
6. RESPECTFUL AND FAMILY-CENTERED CARE 47
6.A Respectful and/or Family-centered Care Policies and Training 47
6.B Patient Satisfaction 47
6.C Family Involvement 48
6.D Infrastructure 48

7. INFECTION PREVENTION AND CONTROL....49

7.A Infrastructure 49
7.A.1 Facility Autoclave and Sterilisation 49
7.A.2 Infection Prevention, Detection and Control Protocols 50
7.A.3 Neonatal Unit Hand Hygience Policies and Functionality 50
7.A.4 Neonatal Unit Hand Hygiene Infrastructure 50
7.A.5 Neonatal Unit Hand Hygiene Behaviour 50
7.A.6 Facility and Neonatal Unit Toilets and Latrines 51
7.A.7 Neonatal Unit Sterilisation, Ventilation and Waste Management 51
7.A.8 Staff Personal Items 52

7.B Medical Supplies and Laboratory 52
7.B.1 Infection Prevention Supplies 52
7.B.2 Laboratory Testing and Capacity for Microbiology 52
7.B.3 Laboratory Linkage to Neonatal Unit 55
7.B.4 Other Protocols for Laboratory Cultures 55
7.B.5 Personal Protective Equipment (PPE) 56

IV. APPENDICES....56

Neonatal Unit Layout 56

Biomedical Technician Workshop Layout 56

Laboratory Protocols 56

V. ACKNOWLEDGEMENTS....56

List of Acronyms

**Acronym**	**Expanded Meaning**
**BCG**	Bacillus Calmette Guerin
**CD4**	Cluster of differentiation 4
**CPAP**	Continuous positive airway pressure
**DHIS2**	District health information system
**GP**	General practitioner
**G6PD**	Glucose phosphate dehydrogenase
**HBIG**	Hepatitis B immune globulin
**IM**	Intramuscular
**IV**	Intravenous
**KMC**	Kangaroo mother care
**Ob/Gyn**	Obstetrics and Gynecology
**PMTCT**	Prevention of mother to child transmission

**Term**	**Definition**
Not applicable	Does not apply to this facility
Not asked	This question was not asked at this facility
Not recorded	The response to this question was not recorded at this facility

Summary

Facility Overview

Item	Results
Name of facility	
Type of facility	
Ownership/operating agency	
Facility has a specified catchment area	
Catchment population (hfa_year)	
Urban/rural designation	
Facility in designated hardship area	

Small and Sick Newborn Care Patient Services Provided

Item	Results (hfa_yearprev)
Annual deliveries	
Annual neonatal unit admissions	
Annual neonatal referrals *into* facility	
Annual neonatal referrals *out of* facility	

Facility Score

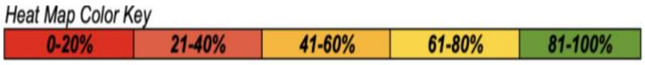

I. BACKGROUND

Purpose

The Every Newborn Action Plan (ENAP) set new coverage targets and milestones for 2020-2025.[1] ENAP focusses on the coverage of antenatal, birth, postnatal and newborn care at national and sub-national levels. The ENAP coverage goal for small and sick newborn care is for 80% of districts in every country to have at least one inpatient newborn care unit by 2025.

In addition, the Sustainable Development Goals set out global targets for health to be achieved by 2030. This includes Sustainable Development Goal 3.2 (SDG 3.2) to reduce live births to less than 12 deaths per 1000 live births globally.[2] However, at the current rates of progress, about 60 low- and middle-income countries, primarily in Southern Asia and sub-Saharan Africa, will not meet this target. 38 of these countries, 74% of which are in sub-Saharan Africa, will need to double their current progress to meet this target.[3]

Newborn Essential Solutions and Technologies (NEST360) alliance (16 organisations, 11 in Africa) is working with governments in Malawi, Kenya, Tanzania, and Nigeria to reduce newborn deaths in hospitals by adopting a co-created health systems package with innovative technologies, mentoring for clinicians and engineers, and evidence-based implementation strategies for sustainability.[4] In order to improve survival, health facilities must be ready to provide high quality care to these small and sick newborns.

Health facility service readiness tools are widely used for measuring basic and comprehensive emergency obstetric care (eg United Nations Population Fund's Improving Emergency Obstetric and Newborn Care tool[5]) or wider service provision (eg World Health Organisation's (WHOs) service availability and readiness assessment[6]). Some of these tools include assessment of readiness to provide basic newborn care, especially in maternity services. However, existing tools do not assess service readiness for inpatient small and sick newborns and have particular gaps in assessing WHO level 2 care including for respiratory support, notably CPAP and for newborns requiring more medical devices, and a higher level of nursing and medical care.

To fill this measurement gap, a health facility assessment (HFA) tool was systematically developed to assess service readiness for small and sick newborn care, building on existing tools and designed to complement tools for maternity care. Co-design of the HFA was facilitated by NEST360, working with African governments and in partnership with United Nations Children's Fund (UNICEF) and other key experts. The HFA was also designed to be interoperable with other quantitative data collected by NEST360 and partners, including quality improvement data. Data will be used to support data-driven decision-making to improve quality of small and sick newborn care.

Methodology

Tool Development

The HFA was developed in 4 stages:

1. Matrix development including items required for small and sick newborn care based on norms and standards, notably WHO’s
2. Mapping existing HFA tool questions against this matrix of required items
3. Co-design of a new HFA tool
4. Refining the HFA, with rollout and learnings in Malawi, Kenya, Tanzania, and Nigeria

Stage 1: Matrix development including items required for small and sick newborn care based on norms and standards, notably WHO’s

Moxon et al. developed a list of over 600 key items for service readiness for inpatient small and sick newborn care.[7] The items were identified from existing international guidelines and standards for small and sick newborn care with a focus on drugs, devices and consumables, and guidelines. We expanded this initial list to include additional items and ingredients necessary for providing care for each of the WHO signal functions for newborn care.[8] New items primarily included detailed data on device availability and functionality, consumable supply availability, and facility and neonatal unit staffing. Existing HFA tools were mapped against this expanded list of items in Stage 2.

The WHO standards for small and sick newborn care were not yet available at this stage.

Stage 2: Mapping existing HFA tool questions against this matrix of required items

We identified 16 existing global and national tools that measure service readiness for small and sick newborn care (Table 1). Identified tools were mapped against the expanded list of items. Individual questions were considered to cover the relevant item if the question was an exact or partial match for the item.

Heat maps were developed to identify patterns showing what proportion of items were included in existing questions in each tool. A conservative threshold of 40% or greater was used to indicate good coverage of items in the tool.

Tools covered a mean of 13.2% (range 2.2-32.7%) of key items. Global tools covered many key items for medicines, lab testing and supplies, and human resources, but had major gaps in neonatal unit infrastructure and information systems. The Improving Emergency Obstetric And Newborn Care (EmONC) and Every Premie tools were found to cover the highest proportion of key items.[5,9] These tools were used as a basis for development of the new HFA tool in Stage 3.

Table 1: List of all service readiness tools identified and mapped

Kenya	KEMRI Project Clinical Service Review and Supervision Tool - Quality of Neonatal Hospital Care
	KEMRI Project Maternity Structure Tool
	KEMRI Project Neonatal Structure Tool (RedCap)
	Kenya Public Health Checklist Forms
	CPHD health facility capacity assessment form
Malawi	CPAP Project Neonatal Care Indicators Checklist
	CPAP Project Ward Assessment Checklist
Tanzania	Star Rating Tool Hospital
	Star Rating Tool Health Center
	Star Rating Tool Dispensary
Nigeria	Appraising Neonatal Care in Tertiary Centers
Global	WHO: Standards for improving the quality of care for small and sick newborns in health facilities
	SPA
	SARA
	EmONC
	Health Facility EmONC/MNCH Assessment Tool 2016
	Every Preemie Scale Facility Assessment for Inpatient Care of Small and Sick Newborn

Stage 3: Co-design of a new HFA tool

Mapping existing tools in Stage 2 identified major gaps in assessment of service readiness for inpatient small and sick newborn care. A new health facility assessment tool was developed to fill this gap through 3 key stages: 1) initial development of the tool according to the expanded item list; 2) pilot testing and line by line review of the new tool in Malawi and Kenya; and 3) restructuring and expansion of tool according to implementation learnings and identification of gaps during stage 2.

Stage 3A: Initial co-design using the expanded item list

The EmONC and Every Premie tools were used as a basis for development of the new HFA tool. Some maternal components of the EmONC were adapted for neonatal care and all neonatal care components from the EmONC were included. Questions were also adapted from the Every Preemie tool. The new tool was restructured to align with the WHO health system building blocks[10].

Initial design was guided by a June 2019 workshop in Nairobi, Kenya with multi-country teams from Malawi, Tanzania, Nigeria and Kenya, including government, clinicians and data experts. The HFA drafted during the workshop was subsequently refined and then shared with 47 additional global experts in neonatal care, and feedback systematically included.

A priori considerations were also taken into account during this stage (Table 2).

Table 2: Feasibility for large-scale use and a priori considerations for development of a new tool measuring service readiness for small and sick newborns.

Assess level 1, level 2 and transition care for small and sick newborns
Complement maternal and obstetric service readiness tools (eg EmONC)
Feasible to conduct at a facility in one day
Linked to WHO norms and standards for small and sick newborns
Organized by WHO health systems building block linking to the NEST Theory of Change
Enabling prompt feedback to facilities and access to full GIS-linked dataset

Stage 3B: Pilot testing and initial review of the new tool in Malawi and Kenya

The paper tool was pilot tested at two central and district hospitals in Malawi by senior Ministry of Health (MoH) staff. After the initial pilot visits, the paper tool was coded into REDCap to streamline data collection and improve data quality. Through adaptation into REDCap, the tool was further refined.[11] The electronic REDCap tool was used for the second pilot training conducted on tablets at 8 central and district hospitals in Malawi. Malawi MoH staff with expertise in clinical care, biomedical device management and maintenance, human resources, and health and data systems, conducted another line-by-line review and role play before the pilot visits.

After two rounds of pilot testing in Malawi, the tool was reviewed and pilot tested in Kenya. The Kenya Committee of Experts (CoE), which includes senior MoH staff, UNICEF Kenya, and other key stakeholders, completed a line-by-line review of the tool and provided feedback. After this initial review, a second line-by-line review was conducted with experts including Kenya MoH staff, Center for Public Health and Development (CPHD) staff, and a team from Uganda. A third and final round of pilot testing was conducted at three county and referral hospitals in Kenya.

Between each round of pilot testing and review, feedback was incorporated. Existing questions were modified, new questions addressing gaps in the tool were added, and sections were restructured to streamline data collection.

Stage 3C: Restructuring and expansion of tool according to implementation learnings

Synthesizing feedback from pilot learnings and review led to restructuring the tool to improve visit flow during data collection and better align with the WHO health system building blocks. During this stage, key gaps in the tool identified through initial feedback were incorporated. These include expanded sections for governance and leadership, information systems, hand hygiene behaviour, and respectful and family centred care. In addition, supplementary modules were developed to assess service readiness components available primarily at higher level central and referral hospitals. These include more substantial sections assessing lab capacity for microbiology, infrastructure of the biomedical technician workshop, and capacity for preventive and corrective maintenance of devices. These expanded and additional sections were developed by adapting existing WHO and other global tools where possible (Table 1), and through consultation and review with experts within NEST360.

The WHO standards for small and sick newborn care were released during this stage.[12] A line-by-line comparison of the WHO standards and new HFA tool was conducted, and any key gaps in the new HFA tool were addressed.

Stage 4: Refining the HFA, with rollout and learnings in Malawi, Kenya, Tanzania, and Nigeria

This final stage of development involved additional line-by-line reviews with experts, including many mid-level and senior MoH staff in Malawi, Kenya, Tanzania and Nigeria.

After numerous rounds of testing, review and feedback, subsequent line-by-line reviews produced minimal requests for changes. Answer options were noted as appropriate across all country contexts and key gaps in the tool had been addressed.

Training and Data Collection

A four-day training was conducted for 10 representatives from within the Ministry of Health to serve as assessors for the HFA. The trainees had the following expertise: nursing, clinical and technical (engineering, laboratory and pharmacy, health information systems). Assessors were provided with training about the HFA tool content and structure, data quality, tablet use and REDCap software. Assessors conducted pilot visits at central and district hospitals in Malawi and Kenya.

HFA data collection was completed in one day at each site. Data were collected offline using the mobile REDCap application on Android tablets. Data quality and consistency checks were built into the REDCap projects through skip-logic and validation. Data were subsequently synced to the web-

based REDCap server at the end of each visit where additional data quality checks were completed. Data collected on paper were later entered to the web-based REDCap server by a data clerk in the central office.

Data Analysis

All the datasets are stored on servers of the designated country partner during data collection and post data collection. Data cleaning and verification took place at multiple stages. Initial data verification was completed by country teams after tablet-based collection and after syncing to the web-based REDCap server. Data were primarily checked for completeness and illogical responses at this stage.

Subsequently, data quality and cleaning scripts were developed in STATA for each HFA module. These scripts include finding empty fields, identifying and removing duplicate records, correcting structural errors, including typos and capitalisation errors, and cross-checking variables to look for implausible associations and inconsistencies. Missing data were assessed and recoded as appropriate. Variables were grouped and recoded, and new summary variables were derived to streamline data analyses.

Descriptive analyses were organised by WHO health systems building blocks using the facility as the unit of analysis. A 3-page summary report with key data was generated from the descriptive analyses (see summary below).

References

1. WHO & UNICEF. The Every New Born Action Plan. Ending Preventable newborn deaths and stillbirths by 2030. 2020;(July). https://www.unicef.org/media/77166/file/Ending-preventable-newborn-deaths-and-stillbirths-by-2030-universal-health-coverage-in-2020–2025.pdf.

2. Nations U. SDG Goal 3: Ensure healthy lives and promote well-being for all at all ages. https://sdgs.un.org/goals/goal3.

3. Hug L, Alexander M, You D, Alkema L. National, regional, and global levels and trends in neonatal mortality between 1990 and 2017, with scenario-based projections to 2030: a systematic analysis. *Lancet Glob Heal*. 2019;7(6):e710-e720. doi:10.1016/S2214-109X(19)30163-9

4. NEST360°. NEST360° Newborn Essential Solutions and Technologies: Our Plan. https://www.nest360.org/our-plan. Published 2019. Accessed May 10, 2020.

5. Brun M, Monet JP, Moreira I, Agbigbi Y, Lysias J, Schaaf M RN. Implementation Manual for Developing a National Network of Maternity Units - Improving Emergency Obstetric and Newborn Care (EmONC). *United Nations Popul Fund*. 2020.

6. World Health Organization. Service Availability and Readiness Assessment (SARA): An annual monitoring system for service delivery - Reference Manual, Version 2.2. *Heal Stat Inf Syst*. 2015. https://apps.who.int/iris/bitstream/handle/10665/149025/WHO_HIS_HSI_2014.5_eng.pdf.

7. Moxon SG, Guenther T, Gabrysch S, et al. Service readiness for inpatient care of small and sick newborns: what do we need and what can we measure now? *J Glob Health*. 2018;8(1):1-18. doi:10.7189/jogh.08.010702

8. World Health Organization. *Survive and Thrive: Transforming Care for Every Small and Sick Newborn*. (Kak L, Lawn JE, Lincetto O, Murphy G, Robb-McCord J, Zaka N, eds.). Geneva: World Health Organization; 2019.

9. Every Preemie Technical Materials. https://www.everypreemie.org/technical-materials/.

10. WHO. Monitoring the Building Blocks of Health Systems : a Handbook of Indicators and their Measurement Strategies. 2010:110.

11. Harris PA, Taylor R, Thielke R, Payne J, Gonzalez N, Conde JG. Research electronic data capture (REDCap)—A metadata-driven methodology and workflow process for providing translational research informatics support. *J Biomed Inform*. 2009;42(2):377-381. doi:https://doi.org/10.1016/j.jbi.2008.08.010

12. WHO. *Standards for Improving the Quality of Care for Small and Sick Newborns in Health Facilities*.; 2020. https://www.who.int/publications/i/item/9789240010765.

I. HFA BASELINE SUMMARY REPORT

Infrastructure

Neonatal Unit Capacity		Designated Areas	
Number of cots		Designated area for high risk/acute babies	
Neonatal unit capacity (including cot/radiant warmer/incubator)		Designated area for low risk/stable babies	
Number of babies in neonatal unit on day of HFA visit		Inborn isolation area	
% bed capacity filled		Outborn isolation area	
Frequency of more than 1 baby per cot		Neonatal admissions only to neonatal unit or KMC	
Frequency of more than 1 baby per radiant warmer/incubator		Area for examination and triage when newborn first arrives	
**Electric Power**			
Fuel generator available in facility		Facility connected to electricity grid	
Fuel generator functional		Stable power (no interruption) for last 7 days	
Battery inverter available in facility		Lighting in neonatal unit covered by backup power	
Battery inverter functional		Equipment in neonatal unit covered by backup power	
Solar power available in facility		No equipment damaged from electricity in last year	
Solar power functional			
**Electricity Safety**		**Fire Safety**	
Floor free of cables (cables not running across floor)		Fire extinguisher available in neonatal unit	
Voltage stabilizer on neonatal unit		Date of last inspection	
Power audit certification in last year		Neonatal evacuation plan available	

Respectful & Family Centred Care

Respectful Care		KMC Capacity	
Functioning family toilets available		Number of KMC beds	
Functioning bathing facilities available		Number of KMC beds that sit up	
Mothers/caretakers can visit at any time		Number of KMC reclining chairs	
Caretakers receive formal counselling before discharge		KMC beds meet demand	

Medical Devices and Supplies

Device and Consumable Availability

Digital Scale	Digital Thermometer	Radiant Warmer	Incubator	Phototherapy	Glucometer	Syringe Pump
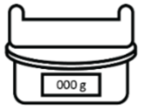	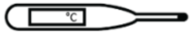	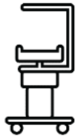	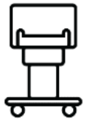	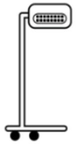	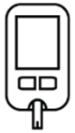	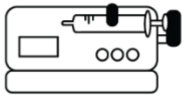
		Probes			*Glucosticks*	
		•			•	

Pulse Oximeter	Suction Pump	Flow Splitter	Bottled Oxygen	Oxygen Concentrator	CPAP
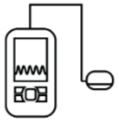	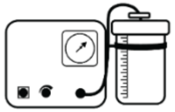	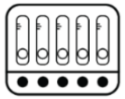	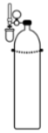	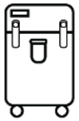	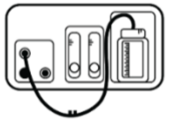
Probes	*Catheters*		*Nasal prongs*		*CPAP prongs*
•	•		•		•
•	•		•		•

Available = available at time of visit, Not available = not available at time of visit, no stockout = no stockout for last 4 weeks

No associated consumables tracked for device

Guidelines	Available	Accessible	Equipment Maintenance	
Thermal protection			Preventive maintenance provided	
Breastfeeding standards			Preventive maintenance system	
KMC practice			Corrective maintenance system	
Oxygen therapy			# Biomed techs/engineers employed by facility	
Fluids, volumes, medications			Biomed techs/engineers employed by facility full-time	
Phototherapy treatment			Biomed present or on-call the night before the HFA visit	

Human Resources

Staffing					
**includes pediatricians and neonatologists* *±exclusively assigned*	Nurse±	Clinical Officer	General Doctor	Spec. Doctor*	Data Clerk±
Assigned to neonatal unit					
On duty at time of visit					N/A
On duty the night before					N/A
Ratio of babies / staff during visit					N/A

Information Systems

Registers and Reporting	
Neonatal unit admissions register used	
Neonatal unit discharge register used	
Electronic summary data submitted (DHIS2)	
**Clinical Audit and Management Meetings**	
Mortality audit/MPDSR team in place	
Mortality audit/MPDSR at least every 3 months	
Quality improvement team in place	
Quality improvement at least every 3 months	

Infection Prevention

Wash			
No regular water shortage in neonatal unit		Waste bin discarded medicines (black)	
Backup water available in neonatal unit		Waste bin infectious (yellow)	
Sinks functioning in neonatal unit		Waste bin anatomical (red)	
Soap or hand sanitizer available in neonatal unit		Sharps container available	
Hand washing wall chart available		Autoclave available	
Hand washing wall chart accessible		Autoclave functioning	
Staff toilets cleaning frequency		Trash bins collected at least once per day	

Laboratory

Laboratory able to perform	
Culture on samples of blood	
Sensitivity on samples of blood	
Culture on samples of CSF	
Sensitivity on samples of CSF	
Serum bilirubin test	
C-reactive protein (CRP)	
Full blood count/examination (FBC/FBE)	

Pharmacy

In stock for last 3 months	
Gentamicin	
Benzylpenicillin	
Amoxicillin	
Ampicillin	
Phenobarbitone	
Caffeine or aminophylline	
Vitamin K	

II. HEALTH SYSTEMS BUILDING BLOCKS

1. INFRASTRUCTURE

1.A Facility Infrastructure

1.A.1 Patient Numbers and Beds

Item	Results
Number of beds in facility (in all departments)	
Number of beds in the labor ward for maternity patients	
Number of beds on the postnatal unit for maternity patients	
Number of beds on the postnatal unit for newborns	
Number of joint maternity and newborn beds on the postnatal unit	
Number of beds in the neonatal unit	
Number of beds dedicated exclusively to neonates in the pediatric unit	
Refused admission or refered a neonate because of inadequate neonatal unit beds in the last year	

1.A.2 Power and Water

1.A.2.1 Available Sources of Power

Item	Fuel Generator	Battery Inverter	Solar Power
Backup power available			
Number of backup power sources available			
Backup power sources functional at time of visit			
Maintenance contract in place			

1.A.2.2 Condition of Power Systems

Item	Results
Facility connected to the electricity grid	
Facility has an energy management plan	
Power outage (exceeding 30 minutes) in the last 7 days	
Sufficient fuel to run the fuel generator at the time of visit	

1.A.2.3 Water

Item	Results
Main water source	
Location of water source	
Reliable backup water supply available	
Main sources of backup water	
Seasonal severe water shortage	

1.A.3 Oxygen Systems, Fire Safety, and Other Infrastructure

1.A.3.1 Fire Safety

Item	Results
Facility conducts regular fire drills (at least once per year)	
Functional accessible emergency exit door available	

1.A.3.2 Oxygen Systems

Item	Results
Walled/piped oxygen available at the facility	
Bulk liquid oxygen tank available at the facility	
Bulk liquid oxygen tank functioning at time of visit	
Oxygen plant available at the facility	
Oxygen plant functioning at time of visit	

1.A.3.3 Sterile Supply Department

Item	Results
Facility provides an onsite or offsite dedicated decontamination area or sterile supply department for medical devices and other equipment	
The dedicated decontamination area or sterile supply department is operated by a licensed decontamination management service	

1.A.4 Physical Areas Available

1.A.4.1 Relevant Physical Areas Available at the Facility

Item	Area present	Water*	Electricity*
*Yes = functioning at the time of the visit; No = not functioning at the time of the visit			
Labor and delivery ward			
Postnatal unit			
General operating theatre			
Maternity theatre			
Special newborn care unit			
Neonatal intensive care unit			
Paediatric ward			
KMC unit			
Laboratory			
Blood bank			
Pharmacy			

1.A.4.2 Newborn care

Item	Result
Neonates admitted to the pediatric ward	
Locations where small and sick newborns receive care	See details below
Dedicated newborn area	
Labour and delivery ward	
Pediatric ward	
KMC unit	
Other:	

1.A.5 Communication Systems and Infrastructure

1.A.5.1 Communication Methods Available for Referrals

Item	Available & Functional	Used for Referral
Landline telephone in the maternity area		
Landline telephone in the neonatal area		
Landline telephone elsewhere in facility		
Cell phone (owned by facility)		
Cell phone (provided to individual staff)		
Public telephone in the vicinity		
Two-way radio		
Internet connection in the maternity area		
Internet connection in the neonatal area		
Internet connection elsewhere in facility		

1.A.5.2 Communications Infrastructure

Item	Results
Dependable cell phone signal at the facility	
Dependable internet signal at the facility	
Facility has access to a closed user group (CUG) system	
Facility has policy to reimburse staff who use their cell phones for work-related calls	
Staff reimbursed by facility for work-related calls	
Locations with functioning computer available	See details below
Neonatal unit	
KMC unit	
Materinity ward	
Postnatal unit	
Paediatric ward	
None of the above	

1.A.6 Transportation

1.A.6.1 Transport for Referral

Item	Results
How newborns are transferred into this facility from another facility	
How newborns are transferred out of this facility to another facility	
Estimated distance to nearest referral facility (in kilometers)	
Estimated time required to drive to the nearest referral facility (in minutes)	

1.A.6.2 Available Modes of Transportation

Item	Total Available	Total functional
Motored vehicle ambulances (a four wheeled vehicle e.g. car, truck, lorry)		
Motorized tricycle ambulances		
Motorcycles		
Bicycle ambulances		
Motorized boats		
Non-motorized boats (e.g. paddle boat, canoe)		
Animal drawn carts		
Stretchers (not part of ambulance)		
Other:		

1.A.6.3 Supportive Infrastructure for Motor Vehicles

Item	Results
Routine preventive maintenance schedule available	
Person responsible for providing corrective maintenance to motor vehicles when needed	
Funds available today for maintenance/repair if needed	
Fuel management plan available	
Sufficient fuel available to transport newborns today	
Driver available to transport newborns today	
Nurse/paramedic available to transport newborns today	

1.B Neonatal Unit Infrastructure

1.B.1 Neonatal Unit Capacity and Transfer

1.B.1.1 Neonatal Unit Capacity

Item	Results
Frequently = more than 2 times/week; Sometimes = less than once/week; Rarely = less than once/month; Never = less than once/three months	
Neonatal care provided 24 hours a day/7 days a week	
Capacity of neonatal unit if 1 baby per cot, incubator or radiant warmer	
Number of cots	
Number of mobile baby cots with castors	
Number of functional mobile baby cots with castors	
Number of stationary baby cots without castors	
Number of functional stationary baby cots without castors	
Mattress available for every baby cot	
Number of mattresses available	
All baby cots have insecticide treated bednets	
Distance between the closest two cots (in centimeters)	
Number of babies currently in unit	
Percent neonatal unit capacity filled	
Percent neonatal unit beds filled	
More than one baby per cot	
More than one baby per radiant warmer	
More than one baby per incubator	

Item	Results
More than one baby per phototherapy light	
Number of babies currently under phototherapy lights in the neonatal unit	
Percent available phototherapy lights filled	

1.B.1.2 Neonatal Transfers

Item	Results
Neonatal transfer method from the labor ward to the neonatal unit	See details below
Carried by hand	
Carried by KMC	
Transferred in an incubator/radiant warmer	
Transferred in a cot	
Other transfer method:	
None specified	
Neonatal unit is in the same building as the labor ward	
Walk time from labor ward to neonatal unit	
Neonatal unit is in the same building as the maternity theater	
Walk time from maternity theater to neonatal unit	

1.B.2 Neonatal Unit Dedicated Spaces

Item	Results
*Adequate space means there is enough space for all visitors.	
**Neonatal Unit Areas**	
Areas available in neonatal unit	See details below
High risk/acute area	
Low risk/stable area	
Area for those admitted outside the facility	
Isolation room	
Dedicated area for exam and triage upon newborn arrival	
Other:	
**Nurses Station**	
Nurses station or staff work area within the neonatal unit	
Place for staff to sit (e.g. chair, bench)	
Nurses can see every baby from the nurses' station	

1.B.3 Neonatal Unit Temperature, Lighting, Oxygen Systems, Water and Fire Prevention

Item	Results
**Neonatal Unit Temperature**	
Functional heating arrangements in neonatal unit	
Electric space heaters	
Space heaters placed appropriately	
Functional room thermometer within neonatal unit	
Number of rooms within the neonatal unit that have functional room thermometers	
**Ambient room temperature (°C) on day of visit**	
Temperature of Room 1	
Temperature of Room 2	
Temperature of Room 3	
Temperature of Room 4	
Temperature of Room 5	
Temperature of Room 6	
Temperature of Room 7	
Temperature of Room 8	
Temperature of Room 9	
Temperature of Room 10	
Draught observed coming from any windows	
Comments on draught from any windows	
**Lighting**	
Adequate lighting during the day	
Adequate lighting at night	
**Oxygen**	
Oxygen wall outlets/ports in the neonatal unit	
Number of oxygen wall outlets/ports in the neonatal unit	
Walled/piped blended oxygen/medical air in neonatal unit	
**Water**	
Routine water shortage in neonatal unit	
Reliable backup water supply available	
**Fire Prevention and Emergency Drills**	
Fire extinguisher available	
Fire extinguisher ever formally inspected for functionality	
Date of last inspection	
Facility has a neonatal evacuation plan in case of a fire	
Emergency care drill on the neonatal unit in the last 12 months	

1.B.4 Neonatal Unit Electricity and Power

1.B.4.1 General Electricity and Power

Item	Results
Sufficient outlets: all equipment can be plugged in without unplugging something else. Adequate means staff are able to easily carry out work.	
Power outages are impacting care on the neonatal unit	
**Outlets and Power Strips**	
Sufficient electrical outlets in unit	
Number of electrical outlets in unit	
Number of power strips/surge protectors available for use	
Number of plugs among all power strips/surge protectors that are available for use	
Number of power strips/surge protectors in use	
Number of devices currently connected to any of the power strips/surge protectors	
Devices connected to the surge protectors at time of visit	
Inline voltage surge protector (e.g. AVS) available	
Cables observed on floor that can be tripped over on day of visit	
**Power Quality Assessment**	
Voltage stabilizer available in unit	
Power audit and certification completed in the last year	
Equipment broken in the last year due to electricity problems	
Facility conducts internal power quality assessments	
Facility experiences power surges or lags	
Appropriate power supply amperage given equipment on neonatal unit	
Comments regarding amperage of the power supply	

1.B.4.2 Neonatal Unit Backup Power

Item	Fuel Generator	Battery Inverter	Solar Power
Backup power available			
Neonatal unit covered by backup power			
Backup power exclusively covers the neonatal unit			
Neonatal unit lighting covered by backup power			
Neonatal unit equipment covered by backup power			

1.B.5 Admission and Referral Criteria

Item	Results
**Neonatal Unit Admissions Criteria**	
Criteria for inborn admission to neonatal unit	
Criteria for outborn admission to neonatal unit	
Criteria for admission to neonatal unit by age	
General or specific criteria for admission to neonatal unit	
Specific criteria for admission to neonatal unit mentioned by the respondent	See details below
Birth asphyxia	
Neonatal sepsis	

Item	Results
Congenital abnormalities	
Born before arrival	
Low birth weight <2.5kg	
Macrosomia > 4.5kg	
Respiratory distress syndrome	
Low apgar score	
Suspected meconium aspiration	
Prematurity	
Birth trauma	
Vacuum extraction	
Rashes and septic spots	
Local infections	
Neonatal jaundice	
Hypothermia	
Unable to breastfeed	
Fever	
Prolonged rupture of membranes/maternal sepsis	
Maternal hypertension	
Maternal psychiatric condition	
**Neonatal Unit Discharge Criteria**	
Specific criteria for discharge from neonatal unit mentioned by the respondent	See details below
Stable/no danger signs	
Time stable before discharge	
Finished medications/treatment	
Minimum discharge weight	
Feeding established (eg able to breastfeed)	
Specific bilirubin level	
**Neonatal Admission to Pediatric Unit Criteria**	
Criteria for inborn neonatal admission to pediatric unit	
Criteria for outborn neonatal admission to pediatric unit	
General or specific criteria for neonatal admission to pediatric unit	
Specific criteria for neonatal admission to pediatric unit mentioned by the respondent	See details below
Meningitis	
Neonatal sepsis	
**KMC Unit Admissions Criteria**	
Specific criteria for admission to KMC unit mentioned by the respondent	See details below
Stable/no danger signs	
Minimum admission weight	
Maximum admission weight	
Low birth weight	
Prematurity	
Mothers willing to practice KMC	
Discharged from NICU	
Feeding established (eg able to breastfeed)	
**KMC Unit Discharge Criteria**	

Item	Results
Specific criteria for discharge from KMC unit mentioned by the respondent	See details below
Stable/no danger signs	
Minimum discharge weight	
Mother educated/able to practice KMC	
Feeding established (eg able to breastfeed)	
**Criteria for referring newborn to higher level facility**	
General or specific criteria for newborn referral to higher level facility	
Specific criteria for newborn referral to higher level facility mentioned by the respondent	See details below
Oesphageal atresia	
Congenital abnormalities/birth defects	
Not improving with treatment	
Hydrocephalus	
Bowel obstruction	
Imperforated anus	
Congenital heart disease/heart defects	
IV fluids required	
Blood transfusion required	
Ventilation required	
ECG required	
Echo required	
X-ray required	
Surgery required	
**Criteria for referring newborn to lower level facility**	
Specific criteria for newborn referral to lower level facility mentioned by the respondent	See details below
Orthopaedic	
Stable post-surgery	
Physiotherapy	
Ambulatory KMC	
Continued care at health facility	

2. MEDICAL DEVICES AND SUPPLIES, PHARMACY, AND LABORATORY

2.A Medical Devices and Supplies

2.A.1 Neonatal Equipment & Supplies

Item	Results	
Available = available at time of visit, Not available = not available at time of visit, No stockout = no stockout for last 4 weeks		
**Diapers**		
Baby nappies/diapers napkins (cloth)		
Baby nappies/diapers (disposable)		
**Blood collection equipment & supplies**		
Blood collection tubes (appropriate small size) e.g. vacuum tubes serum and EDTA (3ml and/or 6ml)		
Blood collection tube holders		
Blood collection vacuum tube needles 22G		
Blood pressure (digital) including neonatal sized blood pressure cuff*		
Blood pressure apparatus (manual/analog) including neonatal sized blood pressure cuff		
Blood pressure transducer (for central arterial lines)		
Capillary sample tubes (glass collection tubes)		
**Endotracheal tubes**		
Endotracheal tubes size 2		
Endotracheal tubes size 2.5		
Endotracheal tubes size 3		
Endotracheal tubes size 3.5		
Endotracheal tubes (any size)		
**Electrocardiography**		
Electrocardiogram (ECG) recorder		
ECG leads (reusable)		
ECG electrodes/stickers (consumable)		
ECG electrodes (reusable)		
**Imaging**		
CT scanner (may be shared with other units)		
	Manufacturer:	Model:
Echocardiography		
MRI (may be shared with other units)		
Portable ultrasound scanner with appropriate probes (e.g. for cranial ultrasound) (may be shared with other units)		
X-ray viewer (negatoscope) (may be shared with other units)		

Item	Results	
**Glucometer & supplies**		
Glucometer 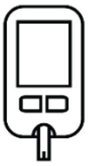		
	Manufacturer:	Model:
Glucometer test strips		
**Weighing scales**		
Weighing scales for newborns (digital) 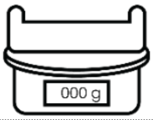		
	Manufacturer:	Model:
Weighing scales for newborns (manual)		
**Other**		
Clean blankets, towels, or linen (for drying baby)		
Dressing trays/procedure trays (or equivalent) (sterile)		
Dressing trolley (or equivalent)		
Flashlight/torch		
Gauze (any)		
Gauze (sterile)		
Hemoglobinometer		
IV cannula (18G or 22G or 24G or 26G)		
Identification bands		
Infantometer (height board or mat)		
Intra-osseous needle (or 22G needles)		
Kidney bowls (polypropylene or stainless steel)		
Lumbar puncture needles (or 23G needles)		
Magill's forceps		
Measuring tape<br>Oxygen tubing		
Padded boards and/or splints for neonates and preterm		
pH monitors		
Recharger for batteries		
Sample collection tubes (pus, cerebrospinal fluid)		
Sepsis diagnostics (bedside only)		
Scissors		
Spacer		
Sterile (low flow) lancet for heel pricks		
Sterile surgical blades		
Stethoscope (neonatal)		
Suture set		
Swabs and/or cotton wool balls		

Item	Results	
Tourniquet (or rubber band for scalp vein)		
Wall clock/timer with second hand		
**Nasogastric tubes**		
Nasogastric (NG) tubes size 3.5 with caps		
Nasogastric (NG) tubes size 4 with caps		
Nasogastric (NG) tubes size 5 with caps		
Nasogastric (NG) tubes size 6 with caps		
Nasogastric (NG) tubes size 8 with caps		
Nasogastric (NG) tubes size 10 with caps		
**Pulse oximetry**		
Pulse oximeter 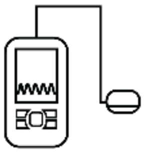		
	Manufacturer:	Model:
Neonatal sized pulse oximetry probes/sensors (reusable, match pulse oximeter)		
	Manufacturer:	Model:
Neonatal sized pulse oximetry probes/sensors (consumable, match pulse oximeter)		
**Suction catheters**		
Suction catheters (size 5)		
Suction catheters (size 6)		
Suction catheters (size 8)		
Suction catheters (size 10)		
**Thermometers**		
Thermometers (digital for newborns that measure 32°C-43°C - must measure below 35.5°C degrees) 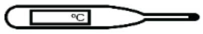		
	Manufacturer:	Model:
Thermometers (analog for newborns that measure 32°C-43°C - must measure below 35.5°C degrees)		
**Umbilical supplies**		
Umbilical cord clamp (cord ties/sterile thread or sterile ligatures or clamp of Barr)		
Umbilical vein catheters size 3.5		
Umbilical vein catheters size 5		
**Urinary equipment**		
Urinary catheter size 3. 4 or 5		
Urine bags (pediatric)		
Urine dipsticks (e.g. Multistix)		
**Patient monitors**		
Patient monitors (at least 3 of: NIBP, HR, SpO2, ECG, RR, Temp) with accessories		
	Manufacturer:	Model:
**Resuscitation equipment & supplies**		
Bag, self-inflating (neonatal size)		

Item	Results	
Neonatal sized face masks (size 0)		
Neonatal sized face masks (size 1)		
Penguin sucker		
T-piece resuscitator		
Resuscitation mannequin		
**Thermal protection equipment & supplies**		
KMC wrappers		
Continuous temperature monitor (not part of radiant warmer)		
	Manufacturer:	Model:
Incubator 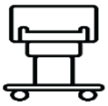		
	Manufacturer:	Model:
Temperature probes (reusable)		
Temperature probes (consumable)		
Radiant warmer 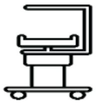		
	Manufacturer:	Model:
Radiant warmer probes		
Embrace warmer		
Heated mattresses (e.g. for hot cots)		
Plastic bags/cling film		
Newborn hats/caps (including preterm sizes)		
Newborn mittens		
Newborn socks		
**Hypoglycemia & IV fluids equipment & supplies**		
Syringe pumps (single phase, delivers microdoses) 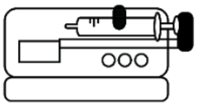		
	Manufacturer:	Model:
Syringes that match syringe pump		
Infusion pumps (delivers larger doses than syringe pump)		
	Manufacturer:	Model:
Adhesive tape		
Neonatal IV tubing/infusion set with burette 100-150ml, sterile, single use		
IV infusion stands without castors		
IV infusion stands on castors		
Sterile needles (19-26 gauge) or butterfly set (23-25 gauge)		
Sterile syringes (size 1; 1cc)		
Sterile syringes (size 2; 2cc)		
Sterile syringes (size 5; 5cc)		
Sterile syringes (size 10; 10cc)		
Sterile syringes (size 20; 20cc)		
Stopcocks 2 or 3 way		

Item	Results	
Rapid blood sugar testing strips/paper reagent strips or equivalent		
**Safe oxygen delivery equipment and supplies**		
Apnea monitor		
	Manufacturer:	Model:
Respiratory rate monitor		
	Manufacturer:	Model:
Oxygen concentrator 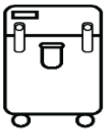		
	Manufacturer:	Model:
	Oxygen output (%):	
Oxygen, bottled/cylinder 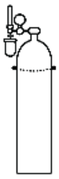		
	Supplier:	
Oxygen flow splitter for newborn 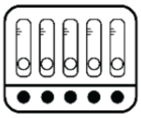		
	Manufacturer:	Model:
Nasal prongs (1 mm)		
Nasal prongs (2 mm)		
Oxygen blenders		
Filters (spare, each type needed for concentrator)		
Oxygen humidifiers		
Oxygen cannula		
**Suction pumps**		
Suction pump (portable, electrical with accessories) 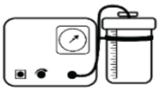		
	Manufacturer:	Model:
Suction pump (manual, non-electric power dependent) 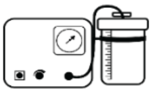		
	Manufacturer:	Model:
**Assisted feeding equipment & supplies**		
Feeding bottles		
Breast pumps (battery powered)		
Manual assisted pumping technology		
Breastmilk substitute		
Breast model to demonstrate how to express milk		
Collection containers (for expressed breastmilk)		
Dolls for nurses to model position and attachment for breastfeeding		
Guide to model position and attachment for breastfeeding (e.g. job aid, video)		
Feeding cups		

Item	Results	
Feeding syringes (any)		
Feeding syringes (sterile)		
Litmus paper/testing strips (or equivalent)		
**Phototherapy equipment & supplies**		
Phototherapy units – LED 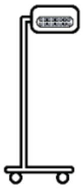		
	Manufacturer:	Model:
	Bulb output:	Bulb last changed:
	Light distance from baby (in cm):	Bulb output if light is 35cm from baby:
Phototherapy units – fluorescent 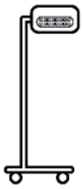		
	Manufacturer:	Model:
	Bulb output:	Bulb last changed:
	Light distance from baby (in cm):	Bulb output if light is 35cm from baby:
Bilirubinometer		
	Manufacturer:	Model:
Test strips for bilirubinometer		
Transcutaneous bilirubinometer		
	Manufacturer:	Model:
Photolight meter and/or phototherapy calibrator		
	Manufacturer:	Model:
Eye patches/eye shields		
Exchange transfusion sets (may be any unit)		
Icterometer		
**CPAP equipment & supplies**		
CPAP driver system with accessories 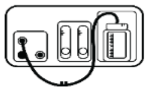		
	Manufacturer:	Model:
CPAP tubing		
CPAP nasal canula		
CPAP prongs (size 000)		
CPAP prongs (size 0)		
CPAP prongs (size 1)		
CPAP prongs (size 2)		
CPAP hats		
Distilled water		
Kettle for boiling water		

Item	Results	
**Mechanical ventilation equipment & supplies**		
Respirator/ventilators plus accessories		
	Manufacturer:	Model:
Portable x-ray (may be shared with other units)		
Hand-held blood gas analyzer (eg iSTAT machine)*		
Cartridges for hand-held blood gas analyzer (e.g. iSTAT, match gas analyzer)		
Infant Laryngoscope (size 0 or 1, straight blades)*		
Laryngoscope light bulb (spare)		
Transilluminators		
Chest drain set		
Drainage tubing and under water seal drainage bottle plus accessories		
CO_2 detector (capnometer)		
**Retinopathy of prematurity equipment & supplies**		
Indirect ophthalmoscope (with small pupil adjustments)		
Condensing lenses 20D for indirect ophthalmoscope		
Condensing lenses 28D for indirect ophthalmoscope		
Neonatal lid speculums (Alfonso)		
Scleral depressors (Schoket/wire vectis)		
Solution for corneal wetting (e.g. Ringers lactate)		
Laser (may be shared with other units)		
Portable diode/green laser with indirect delivery system (may be shared with other units)		
Laser goggles		
**Therapeutic Hypothermia**		
Cooling mattress or Mira cradle*		

2.A.2 Maintenance and Repair

2.A.2.1 Repair: Neonatal Unit

Item	Results
Dedicated technician available for repairing equipment at facility	
Method for contacting technician when equipment breaks	
Time for technician to arrive when equipment breaks	
Confidence that equipment will be returned after giving it to a technician	
Frequency of maintenance provided by the technician on equipment in the neonatal unit	
Maintenance schedules labeled on the oxygen concentrators	

2.A.2.2 Repair: Biomedical Workshop

Item	Results
Inventory list of all equipment available	
Technician employed by	
Repair of medical equipment free for the facility	
Spare parts for broken equipment funded by	

Item	Results
Biggest barrier to repairing equipment	

2.A.2.3 Maintenance Services Provided and Training

Item	Service provided at this facility	Technician trained during pre-service training	Technician has taken a course	Technician has received on the job training
Repair of CPAP device				
Repairing Oxygen Concentrators				
Providing Routine Maintenance on Oxygen Concentrators				
Repairing Phototherapy Lights				
Providing Routine Maintenance on Phototherapy Lights				

2.A.2.4 Biomedical Technician Workshop Spare Parts

Item	Results
Dedicated workshop area available	
Personal protective equipment (PPE) available in the workshop	
**General spare parts available**	
Power cables	
9V Batteries	
AA batteries	
AAA batteries	
Fuses (various)	
**Spare parts available for glucometers**	
Batteries	
**Spare parts available for pulse oximeters**	
Neonatal sized pulse oximetry probe (reusable, clip)	
Neonatal sized pulse oximetry probe (reusable, wrap)	
Recharger for batteries	
**Spare parts available for radiant warmers**	
Temperature probe	
Circuit board (control)	
Circuit board (heating element)	
Power supply unit	
LCD	
Heating element	
**Spare parts available for for incubators**	
Circuit board (control)	
Heating element	
Temperature probe	
Fan	
Control panel (membrane or LCD)	
**Spare parts available for oxygen cylinders**	

Item	Results
Pressure regulator	
Stop valve	
Cap	
Pressure gauge	
Flowmeter	
Pigtail with Safety Wire	
**Spare parts available for oxygen concentrators**	
Flowmeter	
Circuit board (control)	
Circuit board (oxygen monitoring)	
Circuit board/step down transformer (fan)	
Fan	
Solenoid valves (4-way)	
Solenoid valves (equalization)	
Molecular sieve beds	
Power supply unit	
Intake Filter (internal, fine particle)	
Intake Filter (external, gross particle)	
Resevoir tank	
Muffler with exhaust filter	
Compressor	
Compressor rebuild component	
Starting capacitor	
**Spare parts available for oxygen flow splitters**	
Flowmeter	
**Spare parts available for suction pumps (portable, electrical with accessories)**	
Pump assembly	
Vaccuum gauge	
Filter (bacterial)	
Collection reservoir	
Collection reservoir lid	
Power supply unit	
**Spare parts available for suction pumps (manual, non-electric power dependent)**	
Piston assembly	
Vaccuum gauge	
Filter (bacterial)	
Collection reservoir and lid	
**Spare parts available for LED phototherapy lights**	
Circuit board (control)	
Power supply unit	
LED driver/circuit/module	
Control panel (membrane or LCD)	
**Spare parts available for fluorescent phototherapy lights**	
Fluorescent bulb	
Power supply unit	
**Spare parts available for CPAP driver system (standard or bubble CPAP) with accessories**	
Pump assembly	

Item	Results
Filter (internal, pump)	
Flowmeter	
O-ring	
Power supply unit	

2.A.2.5 Biomedical Technician Workshop Tools

Item	Results
Digital Multi meter, digital, LED, with accessories; comparable or similar to Fluke brand piece	
Penlight torch piece	
Pocket magnifier piece	
160 mm combination pliers (heavy duty) piece	
Junior hack saw and blade piece	
Hammer, soft-faced; 1 lb. piece	
Hammer, ballpein; 1 lb. piece	
Soldering station with iron; 240 Vac, 300-500 watts	
Soldering wire	
Soldering tip cleaner (solution or wire sponge)	
Desoldering pump or sucker	
Solder braid wick	
Imperial hex (Allen) key set	
Tape measure; 3 meters; metal, metric piece	
Metric hex (Allen) key set	
Parallel tip screw driver; 6 x 3/16 in. piece	
Flared screw driver; 4 x ¼ in. piece	
Diagonal cutter; 130 mm heavy duty piece	
Straight tweezers piece	
Polycarbonate brass trimmer set (7 items)	
Insulated inspection screw driver (165 mm) piece	
Offset blade screw driver (2 x 6 mm) pieces	
Adjustable wire stripper/crimping tool combination; 0.25 to 0.8 mm piece	
Terminal screw driver piece	
1.5 mm slot screw driver piece	
2.5 mm slot screw driver piece	
Combination spanners	
Reversible screw driver (No.1 and ¾ in. Blades) each	
Engineer's files; wooden handle; hand, square, warding, half round, round and three square in plastic wallet set	
Torx (Tx 8, Tx 10, Tx 15 and Tx 20)	
1.5 mm and 2.5 mm ball end drivers	
Snap ring pliers	
1.5 mm and 2.5 mm nut driver	
Ratchet wrench and sockets	
Bevelled cutter; 4 in. piece	
Small snipe nose pliers piece	
Interchangeable Jeweller's screw drivers with 3 flats and 2 crosshead bits set	
4 in. and 6 in. adjustable spanner each	
150 mm utility pliers (heavy duty) piece	
Snap-off blade knife piece	

2.A.2.6 Biomedical Technician Workshop Storage

Item	Results
**Storage location for new equipment**	
Storage location for new equipment available	
Storage location for new equipment free and protected from insects, birds and rodents	
Storage location for new equipment visibly clean at time of visit	
Storage location for new equipment secured	
Ventilation in the storage location for new equipment	
Minimum documentation available for each equipment order	See details below
None	
Date of receipt	
Name and description	
Quantity recieved	
Name and address of supplier	
Expiry date (if applicable)	
Location for decommissioned equipment available	
**Available in the storage location for new equipment at time of visit**	
Pallet storage/appropriate racking	
Temperature and relative humidity monitoring and recording systems	
Fire protection and fighting equipment	
Warning and information signs	
Quality assurance system and record keeping	
Controlled access (e.g. sign in sheet)	
Lighting sufficient to view all racking areas	
6 x 6 m or more free space for orderly packing, loading & unloading of equipment	
Outside space for loading/unloading equipment	
Transport structures (picking trolley, etc.)	
First aid kit	
Adequate PPE (dust mask, safety goggles, etc.)	
**Workshop Space and Organization**	
Maintenance workshop has an independent space	
Device sorting system used	

2.A.2.7 Biomedical Technician Workshop Systems

Item	Results
**Documentation**	
Documentation available in the workshop	See details below
Corrective maintenance job cards	
Planned preventive maintenance job cards	
Stock cards	
Tool requisition register	
Spare parts requisition register	
Equipment/asset tags	
Decommissioning records	
Procurement records	
None	
**Planned Preventive Maintenance**	
Planned Preventive Maintenance System in use	
Wards covered by the Planned Preventive Maintenance System	
Specific staff allocated to each ward (can be assigned to one ward or multiple wards)	
Frequency of scheduled preventive maintenance	
**Corrective Maintenance**	
Corrective Maintenance System in use	
Wards covered by the Corrective Maintenance System	
Staff allocated for corrective maintenance	
Existing referral system in use to send devices offsite for maintenance	
Corrective repairs documented	
**Decommissioning**	
Decommissioning system in use	
Clear, written procedure for decommissioning	
Included in clear written procedure	See details below
Decommission	
Decontaminate	
Eliminate	
None	
Included in decommissioning procedure	See details below
Request to decommission (user/biotech)	
Decommission confirmation (manager/biotech)	
Decommission approver (manager/MOH)	
Clear points of contact at each decommissioning stage	
None	
Included in decontaminating procedure	See details below
Disassemble	
Manual cleaning	
Mechanical cleaning	
Disinfect	
None	
Included in eliminating procedure	See details below

Item	Results
Disposal	
Reuse	
Refurbish	
Reprocess	
Upgrade	
Donate	
Sell	
Trade-in	
Reassign internally	
None	
Inventory of decommissioned equipment	
Challenges to decommissioning equipment	
**Equipment Leasing and Servicing Contracts**	
Equipment leasing and servicing contracts in use	
Service provider assessment procedure includes	See details below
Responsiveness to fault (e.g. within 48 hours)	
Prior programme of attendance for servicing equipment	
Timely reports produced	
Timely job cards produced	
Responsiveness to requested interventions/issues	
Responsibility in assessing when necessary	
Responsibility in intervening when necessary	
Appropriate skills transfer provided across devices	
None of the above	
**Electronic Maintenance System**	
Electronic maintenance system in use	
Name of the system	
Electronic maintenance system covers	See details below
Equipment Inventory	
Spare Parts Inventory	
Planned Preventive Maintenance	
Corrective Maintenance Record	
Spare Parts Tracking	
Reporting	
Repair Library	
Decommissioning	
Other	
**Reports**	
Monthly reports developed to report to	See details below
Hospital admin	
Regional/District health officers	
Ministry of Health (national)	
None	
Unknown	
Other	
Infrastructural issues (e.g. power or water interruptions) included in reports	
Equipment maintenance issues included in reports	

Item	Results
Reports organized and available for review	
Date of the most recent report	
**Equipment Procurement**	
Procurement plan in place for medical devices	
Guidelines or a system for receiving equipment donations	
Meetings that include biomedical or maintenance staff	

2.B Pharmacy

2.B.1 Pharmacy Systems

Item	Results
*Up-to-date indicates that five randomly selected cards are up-to-date at the time of the visit.	
Pharmacy/drugstore available	
Pharmacy accessible 24/7	
Major source of medicines	
Minor source of medicines	
Drug inventory register/system available	
Frequency of drug stock inventory	
Up-to-date* drug inventory register/system available	
When drugs are ordered	
Primary source of gloves, syringes, and other medical supplies	
Secondary source for gloves, syringes, and other medical supplies	
Most common cause of delay in delivery of supplies	
Second most common cause of delay in delivery of supplies	
"First-expired-First-out" system for supply management used	
Regularly used mechanisms to ensure expired drugs are not distributed	
Drugs stored away from the ground	
Functioning thermometer indicating room temperature in the pharmacy	
Facility has at least one functioning electric/gas (liquid or compressed) refrigerator	
Facility has at least one functioning solar refrigerator	
Drugs available free of charge to neonates	See details below
Gentamicin	
Benzylpenicillin	
Phenobarbitone	
Amoxicillin	
Ampicillin	
Caffeine or Aminophylline	
Vitamin K	
Prices of drugs publicly displayed anywhere in the hospital	
Stockout of following medicines in last 3 months	See details below
Gentamicin (injection)	
Benzylpenicillin	
Phenobarbitone	
Caffeine or aminophylline	
Vitamin K	
Amoxicillin	

Item	Results
Ampicillin	
Action if drug is out of stock	

2.B.2 Essential Drug Availability at Central Pharmacy

Drug	Availability
Available = available at time of visit, Not available = not available at time of visit, No stockout = no stockout for last 4 weeks	
**Antiretrovirals**	
Azidothymidine/Zidovudine (AZT) (oral)	
Nevirapine (NVP) (oral)	
**IV fluids**	
Dextrose 10% with normal saline	
Dextrose 5% with normal saline	
Dextrose/glucose 10%	
Potassium chloride (KCL) 7.5%, 10%, 15%	
Sodium chloride 0.9%	
Ringer's lactate	
Water for injection	
**Antibiotics**	
Amoxicillin (oral suspension)	
Amoxicillin (injection)	
Amikacin	
Ampicillin (IV or IM)	
Ampicillin (oral)	
Benzathine benzylpenicillin (IM)	
Benzylpenicillin (Penicillin G) (IV or IM)	
Cefotaxime (IV or IM)	
Ceftriaxone (IV or IM)	
Flucloxacillin/cloxacillin (IV/IM)	
Flucloxacillin (oral)	
Gentamicin (IV or IM)	
Isoniazid (oral)	
Meropenem	
Metronidazole (IV)	
Metronidazole (oral)	
Piperazine	
Penicillin G Procaine (IM)	
Procaine benzylpenicillin (IM)	
Tetracycline 1% eye ointment	
Vancomycin	
Antibiotic drops (moxifloxacin/betadine)	
**Anticonvulsants**	
Paraldehyde (rectal)	
Phenobarbital (IV or IM)	
Phenobarbital (oral)	
Phenytoin (IV)	
**Vaccinations**	
BCG	

Drug	Availability
Hepatitis B vaccine	
Oral poliomyelitis	
Tetanus immunoglobulin (HTIG) (IM)	
**Analgesics**	
Morphine (IV)	
Morphine (oral)	
Paracetamol (oral)	
Paracetamol (suppository)	
Paracetamol (injection)	
**Corticosteroids**	
Dexamethasone (IM)	
**Emergency drugs**	
Adrenaline/epinephrine (IV)	
Calcium gluconate 10% (injection)	
Hydrocortisone (injection)	
Naloxone (IV)	
**Other drugs**	
Acyclovir (IV)	
Acyclovir 3% topical eye ointment	
Aminophylline	
Anti-rho (D) immune globulin (injection)	
Artificial teardrops	
Caffeine citrate (oral)	
Caffeine citrate (IV)	
Chlorhexidine digluconate 7.1% gel (delivering 4% chlorhexidine)	
Dilating eye drops (tropicamide 0.5% + phenylepherine 2.5%)	
Ethambutol	
Ferrous fumarate (oral syrup)	
Folic acid	
Fluconazole (IV)	
Fluconazole (oral)	
Furosemide (IV)	
Furosemide (oral)	
Glycerin chip	
Hepatitis B immune globulin (HBIG)	
Human milk fortifier	
Lidocaine solution	
Local aneasthetic eye drops (proparacaine 0.5%)	
Miconazole cream (or equivalent e.g. gentian violet)	
Multivitamin	
Nystatin (oral solution)	
Nystatin cream	
Oral rehydration solution	
Phosphate solution	
Sucrose 30% (oral)	
Surfactant	

Drug	Availability
Vitamin K1 (Phytomenadione) (IM or IV)	
Water based lubricant	
Zinc oxide cream	

2.B.3 Essential Drug Availability on Neonatal Unit

Item	Results
Available = available at time of visit, Not available = not available at time of visit, No stockout = no stockout for last 4 weeks	
**Antiretrovirals**	
Azidothymidine/Zidovudine (AZT) (oral)	
Nevirapine (NVP) (oral)	
**IV fluids**	
Dextrose/glucose 10%	
Sodium chloride 0.9%	
Ringer's lactate	
Water for injection	
**Antibiotics**	
Amoxicillin (oral suspension)	
Amoxicillin (injection)	
Amikacin (injection)	
Ampicillin (IV or IM)	
Benzathine benzylpenicillin (IM)	
Benzylpenicillin (Penicillin G) (IV or IM)	
Cefotaxime (IV or IM)	
Ceftriaxone (IV or IM)	
Flucloxacillin/cloxacillin (IV/IM)	
Gentamicin (IV or IM)	
Meropenem	
Tetracycline 1% eye ointment	
**Anticonvulsants**	
Paraldehyde (rectal)	
Phenobarbital (IV or IM)	
**Analgesics**	
Paracetamol (oral)	
Paracetamol (injection)	
**Emergency drugs**	
Adrenaline/epinephrine (IV)	
Calcium gluconate 10% (injection)	
Hydrocortisone (injection)	
Naloxone (IV)	
**Others**	
Acyclovir (IV)	
Aminophylline	
Caffeine citrate (oral)	
Chlorhexidine digluconate 7.1% gel (delivering 4% chlorhexidine)	
Furosemide (IV)	
Miconazole cream (or equivalent e.g. gentian violet)	
Multivitamin	

Item	Results
Nystatin (oral solution)	
Vitamin K1 (Phytomenadione) (IM or IV)	
Water based lubricant	

2.C Laboratory

2.C.1 Laboratory Systems

Item	Results
Facility has laboratory	
Laboratory operational 24 hours per day/7 days per week	
Laboratory has backup power	
Type of backup power	
Laboratory lighting covered by the backup power	
Laboratory equipment covered by the backup power	
Biosafety guidelines available	
Internal quality control guidelines available	
Standard operating procedures (SOPs) available	

2.C.2 Laboratory Equipment and Supplies

2.C.2.1 Equipment and Supplies For Provision of Donor Blood for Transfusion

Item	Results	
Available = available at time of visit, Not available = not available at time of visit, No stockout = no stockout for last 4 weeks		
**Supplies and Equipment for Provision of Donor Blood**		
Refrigerator for blood bank		
Test tubes		
Microscope slides		
Blood lancets		
Cotton wool		
Test tube rack		
0.9% Sodium chloride solution		
20% Bovine albumin		
Electric centrifuge		
37°C water bath or incubator		
Volumetric pipettes		
Blood typing reagents		
Crossmatching reagents		
**Laboratory Supplies**		
Compound Microscope		
Immersion oil		
Glass rods		
Sink or staining tank		
Measuring cylinder, polypropylene, various sizes		
Wash bottle		
Bottle with buffered water		
Blood culture bottles (adult)		
Blood culture bottle (pediatric)		
Timer clock with alarm		

Item	Results
Rack for drying slides	
Giemsa stain	
Wright stain/Eosin	
Funnel and filter paper	
Methanol	
Refrigerator for laboratory supplies	
Refrigerator types available	
Freezer	
Temperature (°C) of freezer	
Glass containers with lids	
Counting chamber (differential counter)	
Pipette, 5ml	
Pipette, graduated 1.0ml	
Dropping pipette	
Cover slips	
Petri dishes	
Culture media	
Kidney dishes	
Tally counter	
Hemoglobinometer	
Manufacturer of Hemoglobinometer	
Hydrochloric acid solution	
Spectrophotometer (symex, screenplus)	
Microhematocrit centrifuge (manual or electric)	
Balance for weighing reagents	
Heparinized capillary tubes, 75mm x 1.5mm	
Ethanol	
Beakers	
Lugol's iodine solution	
CD4 machine	
Sterile picks/loops	

2.C.2.2 Laboratory Tests

Item	Results*
*Available = available at time of visit; Usually available = usually available but not available at time of visit; Not available - but can be outsourced; Not available	
**Biochemistry tests**	
Blood glucose (e.g. glucometer and test strips)	
C-reactive protein (CRP)	
Electrolytes	
Urea	
Calcium	
Magnesium	
Creatinine	
Serum bilirubin (conjugated)	
Serum bilirubin (unconjugated)	
Cerebrospinal fluid analysis (glucose and protein)	
Liver function testing	

Item	Results*
Blood gas analysis	
Urine dipstick for urinalysis that measures: pH, proteins, glucose, ketones, blood nitrates, leucocytes	
Glucose 6-phosphate deyrogenase deficiency (G6PD) screening	
Albumin	
**Hematology**	
Coagulation profile	
Full blood count (FBC)/Full blood examination (FBE)	
Haemaglobin (haemacue) and or haematocrit testing (erythrocyte volume fraction)	
Method used for the full blood count (FBC)/full blood examination (FBE)	
**TB & Malaria testing**	
TB testing (Tuberculin skin testing)	
TB testing (Ziehl-Neilsen staining)	
TB testing (GeneXpert)	
Malaria testing (blood film microscopy or rapid diagnostic tests for neonatal malaria)	

2.C.3 Blood Bank Testing and Supplies

Item	Results
Blood typing and cross matching	
Coombs test	
Storage of whole fresh blood, including type O	
Storage of Rh-negative blood	
Storage of packed cells	
Storage of fresh frozen plasma	
Storage of fresh frozen platelets	
Blood screening for HIV	
Blood screening for hepatitis B	
Blood screening for hepatitis C	
Blood screening for syphilis	
Blood screening for malaria	
Blood screening for sickle cell	
Blood typing and cross matching protocol	
Blood screening for HIV protocol	
Bags for blood and airway needle for giving blood	
Artery forceps	
Anticoagulant bottles (small)	
Anticoagulant bottles (large)	
Scale for blood collection	
**Blood Availability**	
Units of Type A+ blood ready for transfusion	
Units of Type A- blood ready for transfusion	
Units of Type B+ blood ready for transfusion	
Units of Type B- blood ready for transfusion	
Units of Type AB+ blood ready for transfusion	
Units of Type AB- blood ready for transfusion	
Units of Type O+ blood ready for transfusion	

Item	Results
Units of Type O- blood ready for transfusion	

3. HUMAN RESOURCES

3.A Facility Staffing

3.A.1 Medical Unit Staffing

Item	General Nurse	Specialist Nurse	Medical Dr./(GP)	Neonatologist	Paediatrician	Clinical Officer
Formal training can include HBB, COIN, ETAT+, ENC, EmONC etc.						
Staff: Assigned to neonatal unit						
Staff: Assigned exclusively to neonatal unit						
Locum: Assigned to neonatal unit						
Locum: Assigned exclusively to neonatal unit						

3.A.2 Laboratory Staff General Information

Item	Results
Laboratory manager onsite or offsite	
Number of laboratory technicians currently employed by this facility	

3.A.3 Laboratory Staffing

Shift	Physically present	Staff On-Call
Monday – Friday (Day)		
Monday – Friday (Night)		
Saturday (Day)		
Saturday (Night)		
Sunday (Day)		
Sunday (Night)		
Day of visit		
Night before the visit		

3.A.4 Other Established Positions

Item	Neonatal Clerk	Biomed Tech	Lab Tech
Established positions			

3.A.5 Students rotating in the neonatal unit

Item	Medical Students	Nursing Students	Clinical Officer Students
Number of students rotating in the neonatal unit on the day of visit			

3.A.6 Biomedical Workshop Staffing

Item	Biomed engineer	Med Tech / Biomed	Spare Parts Req. Manager	Tools Req. Manager	Electrician	Plumber	Carpenter	Brick Layer	Painter
Allocated to Maintenance Dpt.									
Currently employed by Maintenance Dpt.									
Exclusively assigned to Facility									

3.A.7 Biomedical Staff Scheduling

Shift	Physically Present	Staff On-Call
Weekday (e.g. Monday – Friday) (Day)		
Weekday (e.g. Monday – Friday) (Night)		
Weekend (e.g. Saturday-Sunday) (Day)		
Weekend (e.g. Saturday-Sunday) (Night)		
Day of visit		
Night before visit		

3.B Policies and Working Conditions

3.B.1.1 Facility Policies and Working Conditions

Item	Results
**Contract and Job Description**	
Length of time respondent has been working at facility	
Respondent on a short term (<1 year) or long term (>1 year) contract	
Last on-the-job training in newborn health	
Facility has a written job description for respondent's position	
Reporting structure for administrative purposes is clear	
Respondent has a copy of the job description	
**Staff Rotations**	
Facility conducts staff rotations in and out of the neonatal unit	
Facility has a policy on staff rotation to different units in the facility	
There was a staff rotation of nurses on the neonatal unit in the last 12 months	
Nurses rotated out of the neonatal unit during the last staff rotation	
Up-to-date daily staff rota detailing the shifts of all clinical staff in the neonatal unit available	
Designated person who develops the staff rota for nurses in the neonatal unit	
Shifts on the neonatal unit	
Hours per day covered by the morning shift	
Hours per day covered by the afternoon shift	
Hours per day covered by the evening shift	
Hours per day covered by the night shift	
Hours worked by respondent in the last 7 days	
Nights in a row worked when last on night duty	
Nights off received after being on night duty	
**Staff Cell Phone Policy**	
Personal cell phone used to talk with patients or other providers	
Estimated out of pocket costs paid to talk with patients or other providers in the last 7 days	
Staff reimbursed for out of pocket costs	

3.C Staff Providing Care on the Neonatal Unit

Shift	Physically present in facility	Provide exclusive care on neonatal unit	On-call on neonatal unit
Weekday (e.g. Monday – Friday) (Day)			
Weekday (e.g. Monday – Friday) (Night)			
Weekend (e.g. Saturday-Sunday) (Day)			
Weekend (e.g. Saturday-Sunday) (Night)			
Day of visit			
Night before visit			

3.D Clinical Care Policies and Guidelines

Item	Results
*Easily accessible refers the nurse being able to retrieve the item quickly	
BEmOC and CEmOC guidelines	
Wall charts/action sequences for neonatal resuscitation (e.g. HBB flowchart, ETAT+ flowchart)	
Neonatal resuscitation guidelines	
Thermal protection guidelines	
PMTCT guidelines	
Standards on immediate initiation and exclusive breastfeeding	
Maternal, Infant, and Young Child Nutrition guidelines	
"Ten steps to successful breastfeeding" or implementation of International Code of Marketing of Breast-milk Substitutes written policy	
Fluid, volume, and medication guidelines	
Prevention and management of hypoglycaemia in those at risk of impaired metabolic adaptation guidelines	
KMC guidelines	
Classification of breathing difficulty and SpO2 thresholds and protocols for oxygen therapy and monitoring	
Assessment, management and prevention of apnoea guidelines	
Infant feeding for the neonatal unit, including enteral feeding volumes by weight and age, IV fluid volumes	
IV fluid volume guidelines	
Fluid balance and feeding chart (fluid input and output)	
Providing vitamin D, calcium, phosphorus and iron supplements for very-low-birth-weight newborns protocol	
Treatment thresholds for phototherapy	
Treatment thresholds for exchange transfusion	
Use of phototherapy units (e.g. positioning of phototherapy lamps, etc.)	
Guidelines on management of newborn convulsions and spasms	
CPAP initiation and weaning protocols	
CPAP wall chart	
Mechanical ventilation guidelines	
Blood transfusion procedural guidelines	
Exchange transfusion procedural guidelines	
Blood transfusion monitoring chart	
Assessment and management of newborns with congenital abnormalities, including for specialist consultation and referral pathways guidelines	
Assessment and management of neonatal encephalopathy in newborns guidelines	
Prevention, screening, documentation and treatment of retinopathy of prematurity guidelines	
Management of congenital syphilis guidelines	
Assessment and management of anaemia in small and sick newborns guidelines	
Prevention, monitoring, detection and management of complications related to health care equipment, devices and practices, including skin erythema, skin breakdown, pressure sores, nasal trauma and tissue injury guidelines	
Prevention and management of ophthalmia neonatorum in newborns guidelines	
Pre-referral management of all small and sick newborns who require referral protocol	

Item	Results
Structured, standard form for written handover of newborns among care teams at shift changes or during transfer among facilities protocol	

3.E Newborn Care Signal Functions

3.E.1 Signal Function Performance

Signal functions performed in the last 4 weeks	Results	Challenges to performing signal function in the last 4 weeks
Wash hands with soap or use hand sanitizer before and after handling each baby		
Supporting mothers to exclusively breastfeed		
Resuscitate newborn with bag and mask		
Provide treatment for hypoglycemia		
Provide support for Kangaroo Mother Care (KMC)		
Provide thermal protection		
Administer oxygen to a newborn		
Provide alternative feeding (breast milk substitute) to the newborn		
Provide phototherapy treatment to the newborn		
Administer antibiotics, guided by culture report		
Administer anticonvulsants		
Administer IV fluids to a newborn		
Provide CPAP treatment to a newborn		
Provide mechanical ventilation to a newborn		
Provide blood transfusion for the newborn		
Provide follow-up for retinopathy of prematurity		

3.E.2 Staff Who Initiate and Provide Each Signal Function

Essential services	Signal function performed	Staff who can initiate care	Staff who can provide care
Resuscitate newborn with bag and mask			
Provide support for Kangaroo Mother Care (KMC)			
Provide oxygen to a newborn			
Provide phototherapy light treatment to a newborn			
Provide antibiotics for neonatal infections			
Provide parenteral anticonvulsants to a newborn			
Provide IV fluids using a syringe pump to a newborn			
Provide CPAP treatment to a newborn			
Provide mechanical ventilation to a newborn			

Essential services	Signal function performed	Staff who can initiate care	Staff who can provide care
Perform blood transfusion for a newborn			

3.F Clinical Experience and Training

Essential services	Trained during pre-service training on how to:	Received a formal in-service course in past 5 years on how to:	Received on the job training in past 5 years on how to:	Provided service at facility in last 4 weeks:
Take measures for infection prevention control				
Provide breastfeeding support				
Resuscitate a newborn with bag and mask				
Provide antiretrovirals for prevention of mother to child transmission (PMTCT)				
Treat a newborn for hypoglycemia				
Provide Kangaroo Mother Care (KMC)				
Provide safe oxygen to newborns				
Provide alternative feeding to newborns				
Provide phototherapy to newborns				
Provide antibiotics for neonatal infections				
Manage newborn seizures				
Provide fluids intravenously to newborns				
Provide treatment for anaemia to newborns				
Provide thermal protection to newborns				
Place newborn on CPAP				
Place newborn on mechanical ventilation				
Provide blood transfusion to newborns				
Provide follow-up for retinopathy of prematurity				
Provide management for neonatal encephalopathy				
Provide treatment for congenital syphilis				
Provide palliative care to newborns				
Provide family centered care				
Classify conditions and diseases in accordance with the International Classification of Diseases (ICD)				

Essential services	Trained during pre-service training on how to:	Received a formal in-service course in past 5 years on how to:	Received on the job training in past 5 years on how to:	Provided service at facility in last 4 weeks:
Legal and medical ethical principles of autonomy, informed consent, confidentiality and privacy				
Complete registers currently used at facility				

4. INFORMATION SYSTEMS

4.A Data Sources

4.A.1 Medical Forms

Type of document	Document Used	Creator	Paper or Electronic
**Birth Forms**			
Birth certificate			
Birth notification			
**Inpatient Forms**			
Inpatient diagnostic index card			
Mother and child booklet			
Inpatient/clinical medical notes			
Nursing notes (Cardex)			
Inpatient unit perinatal chart			
Treatment sheet/chart			
Labor, delivery, postnatal case notes			
Reporting form for adverse events			
Admission form			
Prescription form (CC pathway)			
KMC chart			
Vaccination record (BCG Polio 0)			
Facility/ward transfer form (referral)			
Daily bed return form (occupancy)			
**Death and Discharge Forms**			
Discharge form			
Neonatal death audit form			
Perinatal death audit form			
Death certification form			
Neonatal Death Notification			
Perinatal Death Notification			
**Other Forms**			
Neonatal unit admission and discharge form			
Paediatric admission and discharge form			
Mortality and cause of death book			
Follow-up card			
Burial permits			
Other:			
Other:			
Other:			
Other:			
Other:			

4.A.2 Hospital Registers

Type of document	Used	Creator	Paper or Electronic	All data entry fields complete for last 4 weeks	Register filled up to night before visit
**Labor and Delivery**					
Labor and delivery ward register					
Prevention of mother to child transmission (PMTCT) labor and delivery register					
Helping Babies Breathe (HBB) register					
Neonatal admissions register					
Neonatal discharge register					
Postnatal Register					
Outpatient register under 5 years					
Inpatient register under 5 years					
Death/mortuary register					
Referral/counter referral register					
Kangaroo Mother Care (KMC) register					
Immunization permanent register (EPI)					
Child welfare clinic register					
HIV care and treatment register					
Other:					
Other:					
Other:					
Other:					
Other:					

4.A.3 Hospital Tally Sheets

Type of document	Document Used	Creator	Paper or Electronic
Child health and nutrition information systems (CHANIS) tally sheet for child health welfare clinics			
Under 5-year daily outpatient morbidity tally sheet			
Immunization tally sheet			
Other:			
Other:			
Other:			
Other:			
Other:			

4.A.4 Health Summary Reports

Type of document	Document Used	Creator	Paper or Electronic	Frequency of report compilation
Integrated disease surveillance and response (IDSR) monthly report				Monthly
Integrated disease surveillance and response (IDSR) weekly report				Weekly
Immunization services uptake summary report				
Integrated reproductive and child health summary report				

4.A.5 Supply of Forms and Registers

Item	Results
Sufficient is defined as allowing for the documentation of all neonates in the register with extra space to enter additional patients or a new register to use if the current one is filled.	
Neonatal unit had sufficient supply of paper registers in stock at the time of the visit	
Neonatal unit had a stockout of paper registers in the last 4 weeks prior to the time of the visit	
Neonatal unit had sufficient supply of paper inpatient neonatal forms in stock at the time of the visit	
Neonatal unit had a stockout of paper inpatient neonatal forms in the last 4 weeks prior to the time of the visit	

4.A.6 Register Completion on Weekends and After Hours

Item	Results
Routinely is defined as having a system in place to ensure it is always done, except in the case of an occasional extenuating circumstance.	
Neonatal unit routinely filled on the weekends	
Neonatal unit routinely filled after working hours	
Cadre primarily responsible for filling out neonatal unit register	

4.A.7 Filing systems

Item	Results
**Filing on the Neonatal Unit**	
Filing systems used	
Location where medical records for babies are stored during admission	
Cadres with access to medical records for babies on the neonatal unit	
Cadres with access to the active neonatal admissions register	

4.A.8 Storage of medical records

Item	Results
**Storage of medical records for babies discharged alive**	
Years old records are stored for babies discharged alive	
Location where medical records for babies discharged alive are stored after discharge	
Location onsite where medical records for babies discharged alive are stored after discharge	
Cadres with access to medical records for babies discharged alive after they go to central storage	
Cadre responsible for bringing medical records for babies discharged alive to the central storage	

Item	Results
Cadre responsible for managing/overseeing medical records for babies discharged alive in central storage	
Summarize how medical records for babies are transferred from the neonatal unit to long-term storage	
**Storage of medical records for babies discharged deceased**	
Years old records are stored for deceased babies	
Location where medical records for deceased babies are stored after discharge	
Cadres with access to medical records for deceased babies after they go to central storage	
Cadre responsible for bringing medical records for deceased babies to the central storage	
Cadre responsible for managing/overseeing medical records for deceased babies in central storage	
Summarize how medical records for deceased babies are transferred from the neonatal unit to long-term storage	
**Storage of neonatal admissions register**	
Location where old/inactive neonatal admissions registers are stored	
Cadres with access to neonatal registers after they go to central storage	
Cadre responsible for bringing neonatal registers to the central storage	
Cadre responsible for managing/overseeing old/inactive neonatal registers in central storage	
Summarize how old/inactive neonatal admissions registers are transferred from the neonatal unit to long-term storage	
**Other**	
Logistical challenges with medical records experienced in 4 weeks before the visit	
Baby given a unique record ID at the facility	
When baby is given the unique record ID	
Cadre responsible for generating the unique record ID for a baby during office hours	
How baby and mother's medical records are linked	
How multiple babies medical records are linked to each other	
Documents on the neonatal unit are filed away neatly and the surface spaces (e.g. tables, examination areas) are tidy	

4.B Neonatal Data Clerks

Item	Results
Number of neonatal ward clerks currently employed by the facility	
Days of the week neonatal ward clerk usually works	
Facility has a ward clerk exclusively responsible for the neonatal unit	
Facility has a ward clerk exclusively responsible for the neonatal and maternity unit combined	
It is the nurses' responsibility to perform these duties	
Neonatal ward clerk has received a copy of their job description	

4.C Summary Data for Reporting

Item	Results
Indicators routinely calculated at this facility:	See details below
Institutional delivery rate	
Neonatal mortality rate	

Item	Results
Neonatal mortality rate stratified by weight	
Institutional low birth weight rate	
Stillbirth rate	
Neonatal bed occupancy rate	
Referral rate	
Left-against-medical-advice rate	
When indicators are calculated	
This facility submits summary data electronically (e.g. DHIS2)	
Facility has a DHIS2 office	
Number of staff currently work in the DHIS2 office	
Staff responsible for DHIS2 data entry	
When neonatal data are reported to DHIS2	
Data checked by the nurse in charge before submission	

4.E Mortality Audit and Maternal Perinatal Death Surveillance and Response

Item	Results
Facility has WHO MPDSR or relevant national guidelines	
National tools or WHO "tools for implementation" that the facility is using:	See details below
"Terms of reference for review committee" for MPDSR	
"Sample meeting code of practice"	
Individual death care review/clinical summary forms	
Items from the minimal perinatal dataset that are reported to the national level	See details below
Mother's obstetric history (gravida, parity)	
Mother's medical history	
Mother's age	
Single or multiple pregnancy	
Antenatal care history (number of visits)	
HIV status	
Gestational age (and method of determination)	
Place of delivery	
Date and time of birth	
Attendant at delivery	
Mode of delivery	
Sex of baby	
Birthweight	
Date and time of death (if applicable)	
Type of death (antepartum stillbirth, intrapartum stillbirth, neonatal death)	
Cause of death using ICD-10/11 or ICD-PM	
Standardised cause of death classification used at the facility for neonatal deaths (e.g. ICD-PM)	
Neonatal deaths/stillbirths that occur outside the hospital (e.g. brought dead) or in the community are included in the MPDSR process	
Facility has an MPDSR "champion" or lead	
How MPDSR information is communicated to the sub-national level (e.g. district, county)	
Facility receives feedback from the sub-national level (e.g. district, county) on MPDSR information	

4.F Civil Registration and Vital Statistics (CRVS)

Item	Results
Facility has an onsite Civil Registration and Vital Statistics (CRVS) office/registrar	
Staff responsible for submitting data to the CRVS system	
Facility has written, up-to-date guidelines for registering neonatal death and stillbirths	
Births and deaths notified to the civil authority	
Births and deaths captured in the health management information system (HMIS)	

4.G Electronic Information Systems and Infrastucture

4.G.1 Electronic Information Systems

Patient status	Results	Vendor	Vendor support contract	System backup	Encrypted storage
Patient Administration System (PAS)					
Financial Management Syste m and/or Billing System (FMS)					
Laboratory Information Syste m (LIS)					
Picture Archiving and Commu nications System (PACS)					
Outpatient Electronic Medical Records (EMR-outpatient)					
Inpatient electronic medical re cords (EMR-inpatient)					
Comprehensive Care Clinics (CCC) EMR					
Pharmacy Information System (PIS)					
National Health Information System (NHIS-DHIS2)					

4.G.2 Electronic Information Infrastructure

Patient status	Results
Backup power for Information Systems	
Wired network	
IT support staff	

5. GOVERNANCE AND LEADERSHIP

5.A Target Setting

Item	Results
Facility sets targets to achieve	
Facility sets targets for the neonatal unit to achieve	
Facility targets concerning neonatal health or the neonatal unit	
Facility targets displayed in the hospital	
Where facility targets are displayed in the hospital	

5.B Financing Reports

Item	Results
Facility produces a report of revenue and expenditure	
How often report is produced	
When last report was produced	
Facility has an annual budget of the likely costs the facility will face over the next year	
The most common insurance providers that are seen at the facility	

5.C Staff Absenteeism and Performance

Item	Results
Facility has a system of documenting when clinical staff are absent from work	
Facility has a formal system for recognising or rewarding well performing clinical staff	
Actions taken for poorly performing clinical staff	
Facility has a training plan in place to improve the skills of staff on the neonatal unit going forward	
Individual staff have detailed training plans	
Years individual staff training plans cover	

5.D Inventory and Forecasting of Consumables

Item	Results
Inventory register/system for neonatal unit consumables available	
Frequency of inventory of neonatal unit consumables	
Neonatal unit consumables ordered	

5.E Hospital Quality Improvement Team

Item	Results
The health facility has a quality improvement team.	
The health facility conducts quality improvement meetings at least every 3 months.	
Most recent team meeting date	
Meeting conducted in 3 months before date of visit	
Meeting minutes available for the most recent meeting	

5.F Infection Prevention and Control (IPC) Team

Item	Results
The health facility has an infection prevention and control (IPC) team.	
The health facility conducts IPC meetings at least every 3 months.	
Most recent team meeting date	
Meeting conducted in 3 months before date of visit	
Meeting minutes available for the most recent meeting	

5.G Maternal and Perinatal Death Surveillance and Response (MPDSR) Team

Item	Results
The health facility has a mortality audit/MPDSR team.	
The health facility conducts mortality audit/MPDSR meetings at least every 3 months.	
Are review meetings integrated for maternal, stillbirth and neonatal death audit?	
When did the team last meet?	
Meeting conducted in 3 months before date of visit	
Are meeting minutes available for the most recent meeting?	

5.H Lab Management and Inventory

Item	Results
**Lab Management Team**	
The health facility has a lab management team.	
The health facility conducts lab management meetings at least every 3 months	
Date of last meeting	
Meeting conducted in 3 months before date of visit	
Meeting minutes available for the most recent meeting	
**Inventory and Forecasting**	
Inventory of laboratory supplies kept	
Frequency of inventory of laboratory supplies	
Lab supplies are ordered	

5.I Newborn Services and Price Details

Item	Results
Newborn services provided free at the point of care	
Newborn services are provided free at the point of care	See details below
Provide PMTCT services	
Resuscitation of newborn with bag and mask	
Support for breastfeeding	
Treatment for hypoglycemia to a newborn	
Kangaroo Mother Care (KMC)	
Safe administration of oxygen	
Total parenteral feeding to a newborn	
Phototherapy light treatment to a newborn	
Antibiotics for neonatal infections	
Parenteral anticonvulsants to a newborn	
IV fluids using a syringe pump to a newborn	
Management of neonatal encephalopathy	

Item	Results
Treatment for congenital syphilis	
Treatment for anaemia to a newborn	
CPAP treatment to a newborn	
Mechanical ventilation to a newborn	
Blood transfusion for a newborn	
Follow-up for retinopathy of prematurity	
Pre- and post-neonatal surgical care	
Thermal protection to a newborn	
Palliative care to a newborn	
Treatment for congenital abnormalities	
Treatment for ophthalmia neonatorum	
Treatment for necrotizing enterocolitis to a newborn	
Treatment for intraventricular hemorrhage to a newborn	
Prices of treatments and services are displayed publicly anywhere in the hospital	

6. FAMILY-CENTERED CARE

6.A Respectful and/or Family-centered Care Policies and Training

Item	Results
Unnecessary interventions guidelines	
Guidelines for management of newborns suspected of being maltreated	
Pain assessment and management guidelines	
Minimising separation of newborns from mothers/caretakers guidelines	
Discharge protocol	
Developmental follow-up protocol	
Formal policies (e.g. special grieving area etc.) around supporting families of a child who dies	See details below
Guidelines for staff supporting bereaved families	
Protected grieving area	
Counselling services	
Chaplaincy services	
Parent support groups	
Community support groups	
Written support information (e.g. phone number) for bereaved families	
None	
Other:	
Specific respectful and/or family-centered care trainings for staff	See details below
Training in family-centred care	
Training in Kangaroo Mother Care	
Training in legal and medical ethical principles of autonomy, informed consent, confidentiality and privacy	
Training and refresher sessions in end-of-life and bereavement care	
Training and refresher sessions on screening, protecting and managing newborns with evidence of maltreatment, including neglect and violence	
None	
Other:	

6.B Patient Satisfaction

Item	Results
Mechanisms that exist to voice grievances or provide feedback to the facility	See details below
Anonymous survey	
Suggestion box	
Hotline	
Ombudsman	
Exit survey	
None	
Other:	
Facility routinely performs patient satisfaction surveys on the services offered at the facility	

6.C Family Involvement

Item	Results
Information shared with parents about their child's condition	
Parents receive formal counselling about their child's condition before discharge or a training programme on discharge to help them become confident carers	
Activities family members are allowed to do on their sick neonate whilst in hospital	See details below
Bathing	
Feeding	
Turning	
Changing nappies	
None	
Other:	

6.D Infrastructure

Item	Results
*Adequate space means there is enough space for all equipment and for staff/mothers to move around the equipment when the unit is full.	
**Sitting and sleeping arrangements**	
Rooming-in facility within the neonatal unit with a place (e.g. chair, bench, not on the floor) for mothers to sit available	
Rooming-in facility within the neonatal unit with sleeping arrangements available for mothers	
Rooming-in sleeping arrangements available	
Location where mothers with babies in the neonatal unit are housed	
**KMC**	
Facility has a KMC unit	
Number of beds in KMC unit	
Number of beds in the KMC unit that can sit up	
Number of reclining KMC chairs	
Number of mothers in the KMC unit at the time of visit	
Percent occupancy of KMC beds filled at the time of visit	
Space in KMC unit adequate* for volume	
All KMC unit beds have insecticide treated bed nets	
All KMC unit beds have curtains for privacy	
Location where visitors meet the KMC mothers	
Changes to KMC visitor policy due to COVID-19	
Description of changes to KMC visitor policy due to COVID-19	
**Milk**	
Dedicated area for preparing milk/feeds	
Separate space in neonatal unit for private milk expression	
Refrigerated storage space for expressed milk	
Refrigerated expressed milk is labeled	
Human milk bank available at facility	
**Visitors and waiting areas**	
Dedicated waiting area for visitors that is near or in front of the neonatal unit	
Location of the dedicated waiting area for visitors	
Type of waiting area for visitors	
Seating arrangement available in the waiting area	

Item	Results
Seating is adequate* given the number of visitors in the waiting area at time of visit	
Number of people in the waiting area at the time of visit	
Mothers/caretakers allowed into the unit at all times or only specific times	
Mothers/caretakers allowed into the unit at specific time intervals	
Mothers/caretakers allowed into the unit for specific activities	See details below
None	
Bathing	
Feeding	
When crying	
Changing nappies	
Other:	
Changes to mother/caretaker visiting hours due to COVID-19	
Description of changes to mother/caretaker visiting hours due to COVID-19	
**Other areas for families**	
Specific private space for counseling within the neonatal unit	
Space and equipment for mothers to cook available	
Separate/designated 'eating area' for mothers/caretakers	
Breastfeeding mothers provided with high-quality and nutritious meals	
Bathing facilities for caretakers/guardians to use	
Family members permitted to use the bathing facilities	See details below
Mother	
Father	
Grandmother	
Caretaker/guardian	
Other	
Availibility of rota of times the bathing facilities are cleaned	
Access to laundry/washing facilities for caretakers/guardians	

7. INFECTION PREVENTION AND CONTROL

7.A Infrastructure

7.A.1 Facility Autoclave and Sterilisation

Item	Results
Available = available at time of visit, Not available = not available at time of visit	
Separate autoclave room	
Autoclave with temperature and pressure gauges	
Hot air sterilizer (dry oven)	
Steriliser/pressure cooker, electric	
Steriliser/pressure cooker, kerosene heated	
Sterilisation drum	
Sterilisation drum stand	
Autoclave Tapes	

7.A.2 Infection Prevention, Detection and Control Protocols

Item	Results
**Facility has the following infection prevention guidelines and protocols:**	
Prevention of neonatal infections protocol	
Handwashing wall chart	
Cleaning the neonatal unit guidelines	
Cleaning medical equipment guidelines	
**Facility has the following infection control guidelines and protocols:**	
Infection control guidelines	
Early diagnosis and management of neonatal infections protocol	
Drug doses, dilutions and preparations for the neonatal unit guidelines	
Safe and rational use of antibiotics and other medications based on weight and age guidelines	
Prescription chart	
Care of small and sick newborns in outbreak guidelines	
Accidental needle prick protocol	
**Surveillance**	
Surveillance conducted for device-associated infections (for example, catheter-associated urinary tract infections, central line-associated bloodstream infections)	
Surveillance conducted for hospital-acquired infection outbreaks for neonates	

7.A.3 Neonatal Unit Hand Hygiene Policies and Functionality

Item	Results
**All sinks**	
All sinks functioning (running water, not clogged up, not leaking)	
Liquid soaps been diluted with water	
**Hand hygiene policies**	
Facility has policy for staff to wash their hands with soap before or immediately upon entry to the neonatal unit	
Facility has policy for staff to use hand sanitizer before or immediately upon entry to the neonatal unit	
Facility has policy for visitors to wash their hands with soap and/or use hand sanitizer before or immediately upon entry to the neonatal unit	

7.A.4 Neonatal Unit Hand Hygiene Infrastructure

Hand hygiene options	Before or immediately upon at the entrance to the neonatal unit	Before entry to each room in the neonatal unit	In each room in the neonatal unit
Hand hygiene options available			
Type of soap available			
How water taps are operated			
Hand drying options available			

7.A.5 Neonatal Unit Hand Hygiene Behaviour

Item	Results
Hand hygiene opportunities observed	
Indications for hand hygiene (proportion)	
Before touching a patient	

Item	Results
Before clean/aseptic procedure	
After body fluid exposure risk	
After touching a patient	
After touching patient surroundings	
Hand hygiene action taken (proportion)	

7.A.6 Facility and Neonatal Unit Toilets and Latrines

Toilets/latrine options	Separate Staff Toilet	Separate Caretaker / Guardian Toilet	Shared Staff and Caretaker / Guardian Toilet
Toilet/latrine available			
Can be accessed at time of visit			
Can be accessed 24 hours per day, 7 days per week			
Visibly clean and functional			
Hand hygiene options available			
How water taps are operated			
Hand drying options available			
Location of nearest toilet to neonatal unit			
Type of toilet/latrine			
How often toilets are cleaned			
Rota of times toilets are cleaned			
Dedicated person whose job it is to clean the toilet			
Family members permitted to use the toilet			

7.A.7 Neonatal Unit Sterilisation, Ventilation and Waste Management

Item	Results
**Sterilisation**	
Area for cleaning and disinfecting supplies and equipment	
Space for sterilization	
Clean storage space on neonatal unit for supplies	
Is the storage space tidy and organized?	
Disinfection buckets available on the day of the visit	
**Ventilation**	
Functioning air filtration system to prevent risk of airborne infection available	
**Waste Management**	
Functioning incinerator (e.g. burn in incinerator, non-burning incinerator, off-site incineration)	
Types of separate covered waste bins are available on the neonatal unit	See details below
Puncture-proofs sharps container,	
Contaminated waste (red)	
Discarded medicines (black)	
Anatomical/human waste (yellow)	
Glass (blue)	
General trash bin	

Item	Results
None	
Other	
Waste bins used appropriately on the day of visit	
Trash collected at least once per day from the neonatal unit	
Designated staff for waste handling	
Storage space for soiled utility anywhere in facility	
Liquid spills or trash on the floor observed on day of visit	
Vermin (mice, cockroaches) or animal feces ever seen in neonatal unit	
Staff rest area (e.g. tea room, etc.) available	
Food allowed in patient areas	

7.A.8 Staff Personal Items

Item	Results
Number of neonatal unit uniforms owned by respondent	
Clothes respondent comes to work in on most days	
Clothes respondent goes home in after shift on most days	
Frequency that neonatal unit uniform is washed	
Place for staff to leave their personal items (e.g. lockers, cubbies, etc.)	
Staff mobile phones allowed in the neonatal unit	
Caretaker/guardian mobile phones allowed in the neonatal unit	

7.B Medical Supplies and Laboratory

7.B.1 Infection Prevention Supplies

Item	Results
Available = available at time of visit, Not available = not available at time of visit, No stockout = no stockout for last 4 weeks	
Soap	
Antiseptics	
Disposable latex examination gloves	
Heavy-duty gloves	
Non-sterile protective clothing	
Bleach or bleaching powder (chlorine)	
Prepared disinfection solution	
Mayo stand (or equivalent to establish sterile field)	
Surgeon's hand brush with nylon bristles	
Mop	
**Disinfectants and Antiseptics**	
Chlorhexidine (7%) gel	
Ethanol	
Povidone iodine	
Alcohol-based rub	
Hypochlorite	

7.B.2 Laboratory Testing and Capacity for Microbiology

Item	Results
Available = available at time of visit; Usually available = usually available but not available at time of visit; Not available - but can be outsourced; Not available	

Item	Results	
**Microbiology**		
Culture on blood samples		
Sensitivity on blood samples		
Culture on pus samples		
Sensitivity on pus samples		
Culture on cerebrospinal fluid samples		
Sensitivity on cerebrospinal fluid samples		
Culture on urine samples		
Sensitivity on urine samples		
Cerebrospinal cell count		
Gram staining		
Stool analysis		
Group B Strep (GBS) screening		
Protocol for reporting antibiogram/antibiotic susceptibility testing		
Group B Strep (GBS) protocol		
**Blood culture**		
Laboratory method used to conduct blood culture		
Plastic or glass blood culture bottles used		
Make of the blood culture bottles		
Culture media used to make blood culture bottles		
Culture additives used to make blood culture bottles		
Quality controls for the reagents are used in the production of the blood culture bottles		
Make of the Pre-prepared/Procured International Blood Culture Bottles		
Quality controls are used for the pre-prepared/procured blood culture bottles		
Automated Continuous Systems/Machines make		
Automated Continuous Systems/Machines model details	Model name:	Model number:
BD Bactec Bottles used for blood culture		
BioMerieux BacT/Alert Bottles used for blood culture		
ThermoFisher VersaTREK Bottles used for blood culture		
Molecular/genomic machine used	Make:	Model:
Blood culture protocol available		
**CSF culture**		
Method used to concentrate the sample before the CSF culture		
Laboratory method used to conduct CSF culture		
Cultures inoculated directly to an agar plate or into a culture bottle		
Culture media used to conduct CSF primary culture		
Culture additives used to produce the primary agar plates/culture bottles		
Quality controls for the reagents used in the production of the CSF cultures		

Item	Results	
Plastic or glass CSF culture bottles used		
Make of the culture bottles		
Culture media used to make CSF culture bottles		
Culture additives used to make CSF culture bottles		
Quality controls conducted for the locally produced CSF culture bottles		
Pre-prepared/procured international CSF culture system used		
Quality controls conducted for the pre-prepared/procured CSF culture bottles		
Automated continuous systems/machines used	Make:	Model number:
BD Bactec bottles used for CSF culture		
BioMerieux BacT/Alert Bottles used for CSF culture		
ThermoFisher VersaTREK Bottles used for CSF culture		
Molecular/genomic machine used	Make:	Model number:
CSF culture protocol available		
**Identification of Blood Isolates**		
Specific microorganism detected from the blood culture bottle is identified		
Methods used for identification of the microorganism cultured from blood culture bottles		
Media used to subculture blood isolates		
Stains conducted on blood isolates		
Tests used for manual biochemical identification of isolates from blood culture		
Stockouts occured for the reagents used in any of these methods in the 4 weeks before the day of visit		
Comments about the reagent stockouts		
Commercially available assays/minisystems (e.g. API BioMerieux Test Strip etc.) is used for blood culture	Make:	Model:
Stockouts occured for the reagents used in any of these methods in the 4 weeks before the day of visit		
Comments about the reagent stockouts		
**Identification of CSF Isolates**		
Specific microorganism detected from the CSF culture bottle is identified		
Methods used for identification of the microorganism cultured from CSF culture bottles		
Media used to subculture CSF isolates		
Stains conducted on CSF isolates		
Tests used for manual biochemical identification of isolates from CSF culture		
Stockouts occured for the reagents used in any of these methods in the 4 weeks before the day of visit		
Comments about the reagent stockouts		
Commercially available assays/minisystems (e.g. API BioMerieux Test Strip etc.) is used for blood culture	Make:	Model:
Stockouts occured for the reagents used in any of these methods in the 4 weeks before the day of visit		

Item	Results	
Comments about the reagent stockouts		
**Antibiotic Sensitivity Testing**		
Method for antibiotic sensitivity/resistance used		
Etest (Antibiotic Gradient Method)	Make:	Model:
Equipment used in MALDI-TOF MS	Make:	Model:
Equipment used in qPCR	Make:	Model:
Equipment used in DNA Microarray/Chips	Make:	Model:
Equipment used in Loop-mediated Isothermal Amplification (LAMP)	Make:	Model:
Stockouts occured for the reagents used in any of these methods in the 4 weeks before the day of visit		
Comments about the reagent stockouts		
Antibiotic sensitivity testing machines used		
Stockouts occured for the reagents used in any of these methods in the 4 weeks before the day of visit		
Comments about the reagent stockouts		
Protocol for blood culture isolate antibiotic sensitivity testing available		
Protocol for CSF culture isolate antibiotic sensitivity testing available		
Current research studies involving laboratory culture, antimicrobial resistance (AMR) or neonatal infections		

7.B.3 Laboratory Linkage to Neonatal Unit

Item	Number requested in 7 days before visit (Lab)	Number requested in 7 days before visit (Neonatal unit)	Number completed in 7 days before visit (Lab)	Number received in 7 days before visit (Neonatal unit)	Challenges to performing the tests
Blood cultures					
CSF cultures					
Bilirubin tests					

7.B.4 Other Protocols for Laboratory Cultures

Item	Results
**Protocols for cultures (Lab)**	
Record keeping system used for the lab	
How blood cultures are labeled	
Lab has a protocol for reporting culture results back to the neonatal unit	
Describe the protocol for reporting culture results to the neonatal unit	
Time it usually takes to receive results for a suspected sepsis test	
**Protocols for cultures (Neonatal unit)**	
Neonatal unit has a protocol for receiving results from the laboratory and adding them to patient files	

Item	Results
Describe the protocol	
Time it usually takes to receive results for a suspected sepsis test from the lab	

7.B.5 Personal Protective Equipment (PPE)

Item	Results
Gloves (disposable)	
Gloves (reusable)	
Face mask (dust/debris)	
Face mask (surgical)	
Face mask (N95)	
Face shield	

III. APPENDICES

Neonatal Unit Layout

Biomedical Technician Workshop Layout

Laboratory Protocols

IV. ACKNOWLEDGEMENTS

We would like to thank the facility staff who participated, the HFA assessors who collected the data, and the London School of Hygiene and Tropical Medicine and Rice University teams who designed the reports.
